# Tooth Shape Controls Stiffness and Food Collection Efficiency in Biomimetic Radular Teeth

**DOI:** 10.3390/biomimetics11040246

**Published:** 2026-04-03

**Authors:** Wencke Krings, Tamina Riesel, Thomas M. Kaiser, Alexander Daasch, Ellen Schulz-Kornas, Stanislav N. Gorb

**Affiliations:** 1 Department of Cariology, Endodontology and Periodontology, Leipzig University, Liebigstraße 12, 04103 Leipzig, Germany; ellen.schulz-kornas@medizin.uni-leipzig.de; 2 Section Mammalogy and Palaeoanthropology, Leibniz Institute for the Analysis of Biodiversity Change, Martin-Luther-King-Platz 3, 20146 Hamburg, Germany; t.kaiser@leibniz-lib.de (T.M.K.); a.daasch@leibniz-lib.de (A.D.); 3 Department of Electron Microscopy, Institute of Cell and Systems Biology of Animals, University of Hamburg, Martin-Luther-King-Platz 3, 20146 Hamburg, Germany; tamina.riesel@studium.uni-hamburg.de; 4 Department of Functional Morphology and Biomechanics, Zoological Institute, Kiel University, Am Botanischen Garten 1–9, 24118 Kiel, Germany

**Keywords:** gastropoda, functional morphology, additive manufacturing, stereolithography, optimal foraging

## Abstract

Understanding how geometry governs interfacial contact and material removal is central to designing efficient bioinspired surface systems. Gastropod radular teeth form natural arrays of microscale cutting elements optimized for repeated interaction with compliant and semi-rigid substrates, yet experimentally validated shape–performance relationships remain limited. Here, we isolate geometric effects on interfacial mechanics using stereolithography-printed biomimetic tooth arrays inspired by the taenioglossan radula of the hard-substrate grazer *Spekia zonata*. Two morphologically distinct tooth types (central and marginal) were systematically varied in cusp and stylus geometry (four variants each), while array configuration, material, and boundary conditions were kept constant. Tooth stiffness was quantified in bending tests as load-induced height reduction. Interfacial performance was assessed using a controlled pull-through assay in agarose substrates of two stiffness levels (0.4% and 0.8%), with continuous force recording and measurement of removed mass. Marginal-tooth geometries were stiffer and consistently removed more substrate than central variants. Although work increased substantially in stiffer gels, removal did not scale proportionally and declined for central teeth, revealing a decoupling between mechanical input and yield. Performance correlated with active engagement rather than work alone, indicating geometry-limited contact regimes. These findings establish geometry-controlled stiffness and engagement as key parameters for efficient abrasive interfaces.

## 1. Introduction

Mollusks are among the most diverse animal phyla, with an evolutionary history extending back to early Cambrian seafloor communities [1,2,3,4]. Gastropods in particular, comprising ~80,000 extant species [5], occupy an exceptional range of marine, freshwater, and terrestrial environments. This ecological success is closely linked to the evolution of the radula, a key molluscan apomorphy and the principal feeding structure in most gastropods.

The radula enables mechanical interaction with foods and substrates across a wide range of material properties (e.g., hardness, stiffness) and structure (for example, smooth vs. rough or compact vs. porous), thereby facilitating trophic diversification and niche specialization.

Across animals, traits mediating the interaction between organism and ingesta—such as beaks in birds, cranial systems in fishes, or teeth in vertebrates—show strong links to the feeding ecology [6,7,8,9,10,11]. In mollusks, the radula fulfils a comparable role. Embedded within the buccal mass, the radula consists of a chitinous non-extensible membrane bearing serial rows of teeth that are deployed over the odontophore during feeding and is constantly secreted and replaced [12,13,14]. Radular teeth can perform diverse mechanical tasks, including scraping, cutting, tearing, and collecting, and their morphologies often relate to broad trophic strategies and substrate use [15,16,17,18,19,20,21,22,23,24,25,26,27,28].

Importantly, radular teeth do not function as isolated tools. During feeding, the radular membrane bends and stretches, allowing teeth to interact mechanically, transferring forces between neighboring rows to form functionally coupled arrays [29,30,31,32,33]. Experimental breaking-stress tests on real radulae showed that these tooth–tooth interactions can markedly boost load resistance by allowing teeth to bend against adjacent rows, forming stiff arrays [34,35,36,37]. This behavior depends on tooth geometry [34,35,36,38,39,40,41,42], gradients in mechanical properties along the tooth [36,39,43,44,45], the hydration state [34,35,36], and supporting membrane and cartilage properties [40,46]. The extent of this “collective effect” appears ecologically relevant: soft-substrate feeders tend to possess highly compliant weakly coupled teeth, whereas hard-substrate grazers exhibit tighter inter-row coupling [34,35,36].

Despite recent advances, direct experimentally grounded links between radular tooth shape and feeding performance remain limited. Most functional interpretations are still largely inferential, as in vivo manipulation of radular morphology is impossible and direct observation of radular mechanics is limited by small tooth size and rapid motion [14,32]. Biomimetic physical models offer a powerful alternative to isolate morphological variables and test causal effects under controlled conditions. Recently, we introduced a biomimetic pull-through assay to quantify how array-level tooth–tooth interaction influences feeding performance [47]. Using upscaled stereolithography-printed models of marginal-tooth arrays from *Spekia zonata* and standardized agarose substrates, we showed that tightly interlocking arrays removed more food from the substrate per unit work and withstood higher loads, whereas widely spaced arrays bent and deflected away from the substrate. These findings established so called array-level coupling as a key determinant of feeding efficiency. Similar limitations apply to *Conus* radulae, where biomimetic radular models recently revealed that tooth performance depends on material response (fish skin puncture resistance) and engagement geometry (harpoon tip morphology) and not morphology alone [48].

Here, we extend our framework [47] by focusing on tooth shape instead of array spacing. Radular performance depends not only on tooth–tooth interlocking but also on stylus geometry, cusp shape, and effective attack angle—features impossible to manipulate experimentally in living gastropods. We use the teeth geometry from paludomid gastropod *Spekia zonata* (Woodward, 1859), a hard-substrate grazer endemic to Lake Tanganyika [44], as a model system. Its taenioglossan radula exhibits a conserved arrangement of central and paired marginal teeth, with central teeth mainly dislodging algae from stones, while marginal teeth collect and transport particles [35,38,40,44,45]. The three-dimensional morphology of its radular teeth is well characterized, enabling controlled abstraction and proper biomimetic design.

To disentangle geometric effects from array-level interactions, we employed upscaled stereolithography-printed tooth arrays in which stylus (base) and cusp morphologies were systematically varied for central and marginal teeth. Array arrangement, material properties, and geometric scale were held constant to isolate the contribution of individual-tooth shape. The tooth stiffness was quantified using bending tests, while the interfacial performance was assessed in controlled pull-through experiments through agarose substrates of two stiffness levels (0.4% and 0.8%). Mechanical work was recorded continuously, and feeding efficiency was calculated as removed gel mass per unit work.

This experimental framework enables direct testing of how small geometric modifications alter substrate engagement, mechanical response, and material removal. By comparing two morphologically and functionally distinct tooth types, we further assess whether geometry–performance relationships are conserved or tooth-type specific. In doing so, the present study extends our previous findings on array-level coupling [47] by introducing a second experimental axis—within-tooth geometry. Together, these approaches establish a multidimensional framework to evaluate how individual tooth shape and collective mechanical interactions jointly determine performance.

We tested three hypotheses:

**H1.**  *Increased tooth stiffness enhances substrate removal, particularly in stiffer substrates*.

**H2.**  *The effect of cusp geometry on removal and efficiency depends on substrate stiffness*.

**H3.**  *Central teeth, owing to their broader cusps and larger contact area, achieve higher substrate removal and efficiency than marginal teeth*.

## 2. Materials and Methods

### 2.1. Overview of Experimental Framework

The experimental workflow follows the general biomimetic pull-through framework established in [47], in which upscaled additively manufactured radular tooth arrays were drawn through agarose gels, while the force and displacement were recorded to calculate work, and the removed gel mass served as a proxy for food collection. In contrast to [47], which manipulated tooth–row spacing to tune the degree of interlocking (collective effect) in marginal-tooth arrays and included breaking tests, the present study uses manipulations within-tooth geometry (stylus and cusp shape) in both central and marginal teeth, while using a fixed array layout designed to observe individual tooth engagement.

We generated biomimetic physical models of *Spekia zonata* central and marginal teeth (Supplementary Figure S1) with systematically varied morphologies. For each tooth type, four stylus variants and four cusp variants were combined (4 × 4 = 16 variants), resulting in 32 tooth-array models (16 central; 16 marginal). Each model consisted of a square plate carrying 16 teeth. Stiffness was measured in a bending test, and functional performance was assessed in pull-through assays through two agarose gel concentrations (0.4% and 0.8%).

### 2.2. Tooth Modeling and Morphological Variants

An existing 3D radula model from [34] was visualized in MeshLab 2022.02 (International Computing Lab, Italian National Research Council-Institute for Information Science and Technology, Pisa, Italy) and reconstructed in Blender 3.4 (Blender Development Team, Amsterdam, The Netherlands) as a simplified geometric template. Central and marginal teeth were abstracted and used as the basis for design of several biomimetic variants of the radulae (Supplementary Figure S1).

For each tooth type, variants were defined by both stylus and cusp shape (central: styli I–L, cusps M–P; marginal: styli A–D, cusps E–H). Variant naming followed a fixed stylus–cusp mapping, and models were numbered 1–16 per tooth type (for an overview of all types, see Supplementary Table S1 and Supplementary Figure S2; for central tooth stylus types, see Supplementary Figures S3 and S4; for central tooth cusp types, see Supplementary Figures S5 and S6; visualizations of each central tooth model can be found in Supplementary Figures S7–S10; for marginal tooth stylus types, see Supplementary Figures S11 and S12; for marginal tooth cusp types, see Supplementary Figures S13 and S14; visualizations of each marginal tooth model can be found in Supplementary Figures S15–S18). The inter-tooth spacing on the plates was interjacent [47] to facilitate observation of individual tooth behavior and to avoid confounding by strong array stiffening (Supplementary Figure S19). Consequently, this design does not aim to experimentally tune the degree of interlocking as in [47] but instead holds array layout constant while varying tooth geometry.

### 2.3. Array Layout, Scaling, and Printing

Each model comprised 16 teeth arranged in a 4 × 4 configuration (Supplementary Figure S19). Teeth were printed at fixed inclination angles relative to the plate (25°). The printed plate size was 7.4 × 7.4 cm, corresponding to an approximate geometric scale of 1:750 based on tooth width comparisons.

Models were prepared in PreForm version 3.43 (Formlabs, Somerville, MA, USA) and stereolithography-printed on a Formlabs Form 3L (Formlabs, Somerville, MA, USA) 3D printer using UV-curable resin (Grey Resin v4, Formlabs, Somerville, MA, USA). The tooth arrays were printed from Formlabs Grey Resin V4 (Formlabs, Somerville, MA, USA), a UV-curable photopolymer resin recommended for the Form 3/3L platform. According to the manufacturer’s post-cure datasheet, Grey Resin V4 has a tensile modulus of ~2670 MPa, a flexural modulus of ~2740 MPa, an elongation at break of ~11%, an ultimate tensile strength of ~67 MPa, and a flexural strength of ~106 MPa. These values indicate a relatively stiff low-ductility material, which is important for interpreting deformation and contact behavior in the printed models.

The plates were oriented at ~20° during printing to minimize the support structures between tooth and plate. After printing, the models were rinsed in isopropanol and sun-cured for approximately one week; then, the support structures were removed. A two-sided clamping holder was used to flatten the plates during testing.

### 2.4. Bending Test: Tooth Stiffness

**Figure 1:**
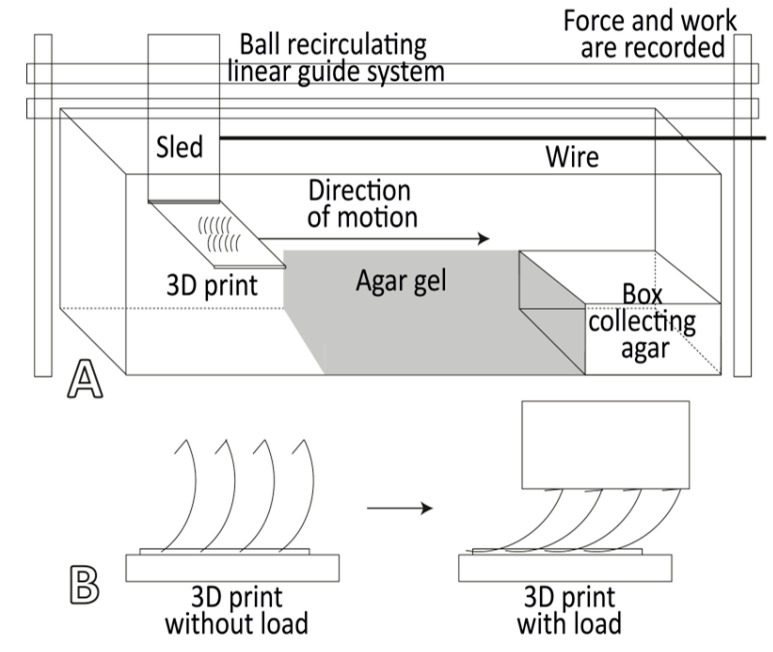
(**A**) Schematic experimental setup of the pull-through test to evaluate efficiency of tooth variants, adapted from [47]. (**B**) Schematic illustration of how tooth stiffness was evaluated with a load.

The loads were set at 400 g (from pilot trials) to cause maximal bending without breaking (Figure 1). Each model was photographed unloaded and loaded. The original tooth height and the height under load were measured from the photos, and the difference was calculated. This was done for each of the four visible outer teeth of each model; we also performed several trails per model. The found differences in height were then used to calculate one mean difference per model. From the load (400 g, 3.92 N) and the height difference (given in m), we calculated the stiffness (given in N/m).

### 2.5. Pull-Through Test

Agarose gels (electrophoresis-grade 604-005, GeneOn GmbH, Ludwigshafen, Germany) were cast at 0.4% (soft) and 0.8% (hard) concentrations in trays following [47]. Tooth arrays were mounted downward, as described in [47], on a Zwicki RetroLine testing machine and pulled at 1 cm min^−1^ through the agar gel (Figure 1). The force was recorded continuously, and the work was calculated over the gel-contact segment (5–21 cm). The removed gel was collected post-assay. The removed gel mass (Δm) was calculated from pre- and post-test gel weights. Each model type was tested once. Empty runs without gel were used to quantify noise from the setup.

### 2.6. Substrate Engagement and Efficiency

After each test, the gels and tooth arrays were photographed. The number of gel-coated teeth and the presence of scratch marks were used as proxies for substrate engagement. The feeding efficiency was calculated as work per unit removed mass (N·mm g^−1^), following [47], where the removed gel mass represents standardized “food” conditions.

### 2.7. Statistical Analysis

All statistical analyses and data visualization were performed in JMP Pro 17 (SAS Institute Inc., Cary, NC, USA). Analyses were descriptive, quantifying shape-dependent variation in mechanical behavior and feeding performance rather than testing manipulated factors.

Data summaries include means, standard deviations, ranges, and rankings. Descriptive comparisons among tooth types (central vs. marginal), stylus types, cusp types, and gel concentrations (0.4% vs. 0.8%) are supported by visualizations.

The feeding efficiency was calculated as the mechanical work per unit removed gel mass (N·mm g^−1^); lower values indicate higher efficiency. Efficiency rankings were generated separately per gel concentration and summed across gel types to provide an integrated measure of overall performance across substrate conditions. These rankings and summed efficiencies served comparative rather than interferential purposes.

## 3. Results

### 3.1. Bending Test: Stiffness

The stiffness values derived from the bending tests are summarized in Supplementary Table S2. Stiffness is reported in N m^−1^, and the sample sizes per variant ranged from two to four trials.

Across all variants, marginal teeth exhibited a higher mean stiffness (466.70 N m^−1^) than central teeth (388.53 N m^−1^). However, the stiffness varied markedly among individual models within both tooth types.

For central teeth, the stiffness ranged from 167.94 N m^−1^ (model 3) and 174.99 N m^−1^ (model 16) to a maximum of 709.25 N m^−1^ (model 6). Other relatively stiff central variants included model 5 (653.66 N m^−1^) and model 13 (626.00 N m^−1^), whereas several models clustered between 200 and 300 N m^−1^ (e.g., models 1, 4, 9, 12, 14).

Marginal teeth showed an even broader stiffness range. The least stiff marginal variants were models 7 (97.76 N m^−1^) and 8 (118.28 N m^−1^), while the highest stiffness was recorded for model 14 (2222.22 N m^−1^), markedly exceeding all other variants. Excluding this outlier, several marginal variants exhibited intermediate to high stiffness values, including model 2 (768.63 N m^−1^), model 4 (714.42 N m^−1^), model 5 (606.15 N m^−1^), and model 13 (493.89 N m^−1^).

Overall, both tooth types displayed substantial morphological variation in stiffness, with marginal teeth showing a larger spread of values and a higher mean stiffness compared with central teeth.

### 3.2. Force, Work, and Removed Gel Mass (ΔM)

**Figure 2:**
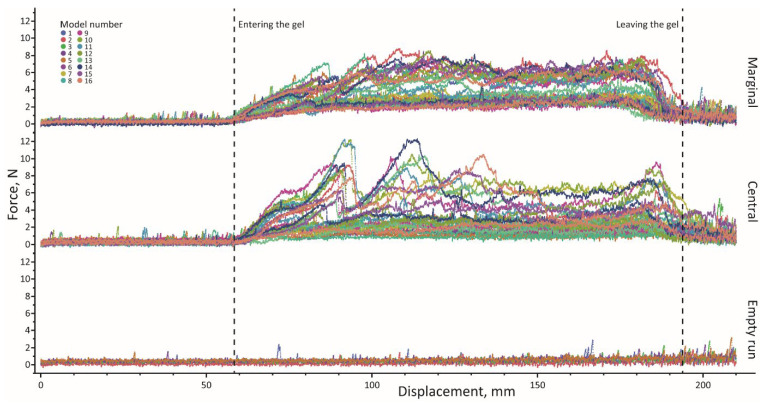
Force–displacement curves showing the full course of the pull-through experiments, grouped by tooth type.

**Figure 3:**
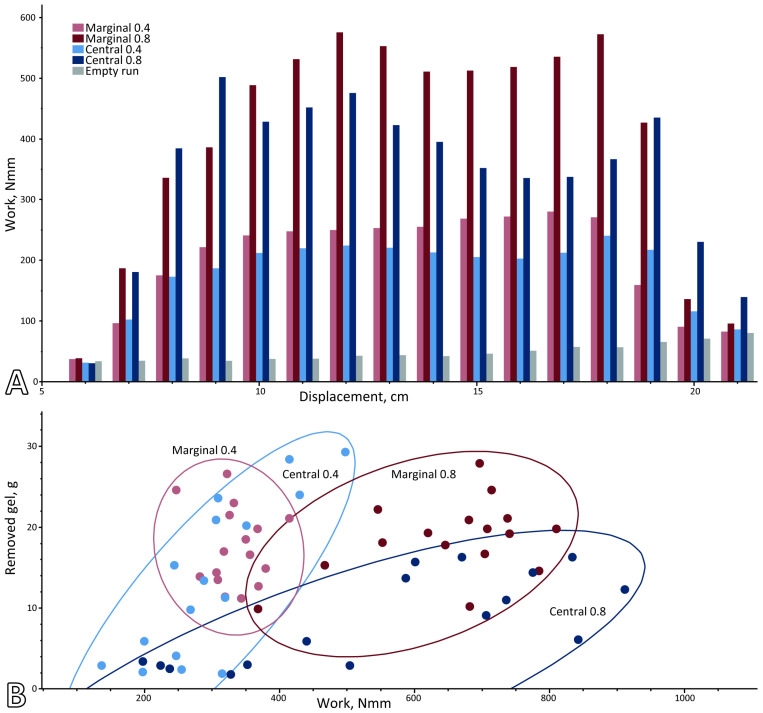
(**A**) Mechanical work per centimeter travelling distance along the section of the pull-through path, where the tooth models interacted with the gel. During the interval in which all tooth rows were engaged (6–20 cm), work per centimeter was relatively constant. (**B**) Summary of pull-through test results grouped by tooth type and gel concentration. Removed gel, given in g, is plotted against work, given in Nmm. Circles represent the scatter ellipses.

The force–displacement curves rose sharply during the tooth–gel contact (Figure 2). Marginal-tooth curves were smoother than those of central teeth, which often exhibited multiple peaks. The work per centimeter increased as the tooth rows entered the gel and decreased as they exited (Figure 3A). This stepwise pattern was especially pronounced in 0.8% gel, where the pre- and post-gel work largely reflected rail friction.

The mean force, work, and Δm for all tooth variants and gel types are summarized in Supplementary Table S3. The tooth types and substrate stiffness both strongly affected the performance. In the softer 0.4% gel, marginal teeth generated low forces (~1.6 N) and moderate work (~170 N·mm), while removing substantial gel (mean 18.1 g, range 11–27.9 g). In the stiffer 0.8% gel, the force and work roughly doubled (3.1 N, ~325 N·mm) but the removed gel mass remained constant (18.1 g), indicating a reduced efficiency on the stiffer substrate. Arrays with the higher stiffness showed greater forces and work but not consistently higher removal.

Central teeth produced lower forces and removed less gel but were more variable. In 0.4% gel, the mean force was ~1.4 N, work ~150 N·mm, and the removed gel mass spanned 1.9–29.3 g. In 0.8% gel, the force and work rose (~2.7 N, ~286 N·mm), while the removal declined sharply (mean 8.4 g); several arrays removed <5 g despite work >400 N·mm, showing decoupling between effort and output.

Empty runs (no gel) yielded much lower forces (0.44 N) and work (49 N·mm), confirming minimal frictional influence from the guide system.

The relationship between the mechanical work and removed gel mass varied strongly with tooth type and gel stiffness (Figure 3B). In the softer 0.4% gel, both tooth types showed wide variation in removed mass at similar work levels, particularly among central teeth, which ranged from high removal at low work to low removal at comparable effort. In the stiffer 0.8% gel, the work increased for both tooth types, but the removed mass did not. Central teeth showed removal despite higher work, clustering at high work but low yield, whereas marginal teeth required more work yet maintained similarity across gels. This indicates a partial decoupling between work and material removal, most pronounced for central teeth in the stiffer substrate.

### 3.3. Δm and Tooth Engagement

The removed gel mass and the proportion of gel-coated (“active”) teeth for all arrays and gels are summarized in Supplementary Table S4. The tooth type, gel stiffness, and engagement levels differed markedly.

Central-tooth arrays showed high variability in both removal and engagement. In 0.4% gel, the removed mass ranged from 1.9 g (model 7) to 29.3 g (model 15), with engagement spanning 43.8% to 100%. Higher engagement generally corresponded to higher removal, though not linearly.

In the stiffer 0.8% gel, the removal declined (1.8–16.3 g), and several models exhibited reduced engagement (25–37.5%). Even high engaged models removed little gel, suggesting that increased stiffness limited effective penetration.

Marginal teeth consistently removed more gel and maintained near-complete engagement across conditions. In the 0.4% gel, the removed mass ranged from 11.2 to 27.9 g and in the 0.8% gel from 9.9 to 24.6 g, with engagement ≥92.3% active teeth in all cases. Their stable removal and high engagement indicate robust and reliable substrate interaction across arrays and stiffness levels.

### 3.4. Effect of Cusp and Stylus Shape

Supplementary Figure S20 presents the removed gel mass, Δm, grouped by stylus types in the upper panels and by cusp types in the lower panels, separately for central and marginal teeth variants and for both gel concentrations.

In the upper panel A (stylus types), central-teeth variants showed pronounced differences in removed mass among stylus variants, particularly in the stiffer (0.8%) gel. Some stylus types exhibited a strong reduction in removal compared with the softer gel, whereas others maintained moderate removal levels. In contrast, marginal teeth displayed comparatively small differences among stylus types, and the removed mass remained relatively consistent between gel concentrations.

In the lower panel B (cusp types), central-teeth variants again showed strong shape-dependent variation. In the 0.4% gel, forward-oriented cusp types removed substantially more material than rounded or curved cusp types, whereas in the 0.8% gel, the removed mass declined across all cusp types. Despite this decline, differences among cusp types remained evident. For marginal-teeth variants, the removed mass was similar across cusp types in both gels, with only minor variation and no consistent ranking among cusps.

This shows that the stylus and cusp shape strongly influenced the substrate removal in central-teeth variants, whereas marginal-teeth variants were comparatively insensitive to both stylus and cusp variation, maintaining stable removal across morphologies and substrate stiffness.

Supplementary Figure S21 illustrates the relationship between the mechanical work and removed mass (*Δ_m_*) for the pull-through experiments, grouped by stylus types on the left panels and cusp types on the right panels, for both central- and marginal-teeth variants. Across all groupings, a clear separation between gel types was evident: experiments conducted in the stiffer (0.8%) gel generally required substantially more work than those in the 0.4% gel, whereas the removed mass did not increase proportionally.

For central-teeth variants, both stylus and cusp groupings showed a wide range of removed mass at comparable work values. Several variants clustered at high work but low removed mass, particularly in the 0.8% gel, indicating inefficient “food” removal from the substrate despite high mechanical investment. Differences among base types were especially pronounced, with some bases showing steep increases in work without corresponding increases in removal. The cusp types also differed in their distribution, with some cusp groups achieving higher removed mass at lower work in the softer gel.

For marginal-teeth variants, the relationship between work and removed mass was more expressed. Across both stylus and cusp types, the removed mass varied within a narrower range, and most data points clustered at moderate to high removed mass values despite increasing work in the stiffer gel. In contrast to the central-teeth variants, marginal-teeth variants rarely exhibited combinations of very high work and very low removal.

### 3.5. Efficiency Ranking

The efficiency rankings confirmed these patterns and revealed strong differences among tooth variants and between tooth types (Supplementary Tables S5–S7). Efficiency was defined as mechanical work per removed gel mass, such that lower values indicate higher efficiency.

Central teeth were highly sensitive to shape, with efficiencies ranging widely (6–94 N·mm g^−1^) and only a few models (e.g., 6, 9, 13) performing well across gel types. The summed efficiencies (Supplementary Table S7) across both gel types emphasized this variability.

Marginal teeth were consistently more efficient (5.07–33.05 N·mm g^−1^), showing less variation and better performance across substrates. Overall, marginal teeth required less work per unit mass removed and maintained stable efficiency regardless of the gel stiffness.

## 4. Discussion

This study builds on emerging biomimetic approaches that translate radular form–function relationships into experimentally tractable physical systems, including soft-robotic concepts inspired by gastropod feeding interfaces [49]. By abstracting radular teeth into standardized upscaled arrays with controlled geometry, such models allow direct causal testing of structure–performance relationships that cannot be manipulated in vivo. Whereas our previous work demonstrated that array-level coupling (the collective effect) strongly enhances feeding efficiency and mechanical robustness in *Spekia zonata* [47], the present study isolates the contribution of individual-tooth geometry. Together, these studies establish complementary experimental axes—collective interaction and within-tooth morphology—that jointly shape radular performance.

### 4.1. Biomechanical Behavior and Stiffness

Effective feeding structures must resist structural failure during repeated loading, as structural damage directly reduces performance and individual fitness. Across biological systems, failure avoidance is achieved through the interplay of geometry, material properties, and load redistribution, as demonstrated for insect wings [50,51], mammalian teeth [52,53,54], or skeletal elements [55]. In radulae, failure involves mechanical support by odontophores and subradular cartilages [56,57,58,59], stress redistribution via chitin fiber orientation in teeth and membrane [46,57,60,61,62], a thin mineral-rich coating on the tooth surface [61,63,64,65,66,67,68,69], and pronounced material gradients along the tooth axis [36,39,43,45].

A distinct feature of many radular systems is that teeth can mechanically support one another during loading. Geometry, orientation, and spacing determine, whether adjacent teeth form stiff load-sharing arrays [29,30,31,32,33,34,35,36]. In our previous biomimetic experiments, increased tooth–row interlocking enhanced both resistance to structural failure and feeding efficiency [47]. Here, the array spacing was kept intermediate (interjacent; see [47]) to isolate the effects of shape. Under these conditions, individual tooth stiffness, based on shape, emerged as a key determinant of performance. Rather than catastrophic failure, the smaller stiffness values manifested primarily as bending and deflection away from the substrate.

In our experiments, marginal teeth were consistently more stable than central teeth mimics. They bent less and maintained contact more reliably than central teeth, likely because their larger height, narrower cusps, and steeper inclination guide force normal to the substrate, promoting better penetration and mechanical stabilization rather than deflection. In central teeth, highly compliant configurations behaved similarly to the non-interlocking arrays described previously [47], yielding under load rather than maintaining effective contact to the substrate. Such behavior resembles radulae of soft-substrate feeders, which favor flexibility, but perform poorly on stiff substrates [34]. Together, both studies indicate that effective feeding on resistant substrates requires controlled stiffness combined with limited compliance, rather than extreme flexibility.

### 4.2. Feeding Performance and Energetic Implications

Across both assays, small geometric modifications caused large changes in mechanical behavior and performance. Marginal-teeth variants were, on average, more stable, more efficient, and less variable than central-teeth variants, falsifying H3. Central-teeth variants exhibited pronounced variability in engagement and removal, indicating strong sensitivity to small geometric changes.

Although the feeding efficiency was quantified here only as the work per removed mass, the results reveal a broader energetic principle: increased mechanical investment does not necessarily translate into increased resource acquisition. This was particularly evident in the stiffer gel, where the work increased sharply, while the removed mass remained constant or even declined, especially for central teeth. Such work–yield decoupling indicates that energy was increasingly dissipated into non-productive processes such as poroelastic compression, friction, sliding, and elastic deformation rather than penetration and cutting.

Our findings on shape-limited performance and work–yield decoupling align with biomechanical patterns observed in *Conus* radulae, where geometry and material properties of target substrate govern interaction outcomes [48]. This system together with our experiments illustrate that engagement geometry and substrate response—not just absolute applied work or force—dictate functional performance in feeding contexts.

From a biological perspective, this suggests that radular morphologies are constrained not only by their ability to withstand force, but by their capacity to convert applied work into productive substrate interaction. Morphologies that fail to achieve reliable engagement incur high energetic costs with little return, which would be disadvantageous under natural feeding conditions. In this context, marginal-teeth variants appear energetically robust, maintaining relatively stable yield across substrates and morphologies, whereas central-teeth variants represent a higher-risk strategy whose performance is strongly contingent on precise geometry and engagement conditions.

### 4.3. Functional Robustness, Specialization, and Mechanical Risk

A central outcome is the marked functional contrast between marginal- and central-teeth variants. Marginal-teeth variants displayed low variance in both removed mass and efficiency across morphological variants, suggesting a degree of functional redundancy and tolerance to morphological variation, wear, or minor positional shifts.

In contrast, central-teeth variants were highly sensitive to small geometric changes. Minor modifications in the cusp shape or stylus geometry shifted central teeth between effective penetration and near-complete functional failure. This sensitivity implies a trade-off between robustness and specialization. Central teeth operate close to a mechanical threshold but allow fine-tuned differentiation when geometry and loading align optimal. This dichotomy aligns with the hypothesized division of labor within taenioglossan radulae [35,38,40,44,45], in which marginal teeth ensure reliable particle collection and transport, whereas central teeth contribute conditionally to loosening or cutting under favorable kinematics and substrate properties.

Viewed through risk-sensitive foraging theory [70,71], this reflects mechanical risk partitioning within the radular system: marginal teeth provide a reliable baseline function; central teeth operate as high-variance component that, while occasionally inefficient, can yield elevated returns under optimal conditions. Such distributed risk management buffers feeding performance against environmental heterogeneity.

### 4.4. Geometry, Deformation, and Contact Mechanics

Higher stiffness generally promoted interaction with the stiffer gel, supporting hypothesis H1. However, the most productive variants were not always the most stable, demonstrating that stiffness alone does not guarantee high removal. This mirrors our earlier findings [47], where strong interlocking enabled stiff cooperative arrays, but effective removal still depended on whether the tooth geometry allowed productive penetration rather than sliding.

The cusp shape influenced performance in a tooth-type-specific manner, which supports H2. In central-teeth variants, forward-oriented cusps (N and M) consistently removed more gel than rounded or curved cusps (P and O), indicating that penetration and cutting dominated under the tested kinematics. In marginal-teeth variants, the cusp effects were weaker and inconsistent across substrates. This contrast is informative in light of [47]: when array-level coupling (ALC) was experimentally enhanced, the feeding efficiency increased strongly even without altering the cusp shape, indicating that collective mechanics can outweigh fine-scale shape effects.

These results suggest trade-offs between immediate efficiency and durability. Less stable teeth may avoid catastrophic failure but dissipate more energy over repeated cycles, whereas stiffer teeth may maintain consistent engagement at the cost of higher local stresses, which was previously also documented in a breaking stress test on real radular teeth [34,35]. Although fatigue and wear were not tested here, the observed deformation behaviors suggest that cumulative performance over multiple feeding cycles could differ substantially from single-pass efficiency, providing an important direction for future work.

### 4.5. Decoupling Work and Yield

Central-teeth variants frequently produced shallow impressions without deep penetration, and only a fraction of teeth became gel-coated, indicating intermittent contact. Broad cusps combined with shallow approach angles likely increased the contact area and promoted poroelastic compression rather than cutting. These limitations were stronger in the stiffer gel, where the resistance to motion increased without a corresponding increase in effective engagement.

In contrast, marginal-teeth variants—taller and narrower—likely generated higher local stresses at the tooth–substrate interface, maintaining penetration and effective scratching across gels and specific variants. This pattern directly complements our previous collective-effect experiments. In [47], tight interlocking produced stiff mechanically integrated working edges that efficiently translated work into removal of the substrate coating. In the present study, interjacent spacing limited array reinforcement. The observed work–yield decoupling therefore reflects the combined effects of geometry-limited penetration and reduced mechanical coupling of neighboring teeth.

The work–yield decoupling observed here is consistent with energy dissipation into non-productive processes at the tooth–substrate interface. Agarose gels are hydrated porous materials, and under load, they can respond not only by local fracture or cutting but also by poroelastic deformation, involving compression of the polymer network together with fluid redistribution within the gel matrix, as generally described for porous and soft-matter systems [72,73,74]. Under such conditions, the applied work was likely not completely converted into substrate removal. These effects are expected to become especially important when the cusp geometry and approach angle do not generate sufficient local stress concentrations for penetration. Thus, the low-yield high-work behavior of several central-tooth variants in the stiffer gel is consistent with a compression- and sliding-dominated interaction regime rather than effective cutting.

The observed decoupling between mechanical work and substrate removal highlights that radular teeth do not simply transmit applied force but instead filter it into distinct mechanical outcomes. Increased work frequently resulted in poroelastic compression, sliding, or elastic deformation rather than productive penetration, particularly in central-teeth variants interacting with stiffer gel. This demonstrates that geometry (cusp shape, width, and effective contact area) strongly determines whether the energy applied to the system is converted into cutting or dissipated non-productively. Consequently, the feeding performance cannot be inferred from the force generation alone; instead, it emerges from the interaction between applied loads and tooth–substrate contact mechanics. This filtering role of shape provides a mechanistic explanation for why increased effort does not necessarily increase yield.

Although efficiency was quantified here for single pull-through events, radulae operate under repeated cyclic loading during feeding. The observed deformation behaviors suggest that cumulative performance may differ from single-pass efficiency: flexible morphologies may avoid catastrophic failure but dissipate more energy over time, whereas stiffer well-engaging geometries may sustain higher local stresses and be more susceptible to abrasion and fatigue, as observed in breaking stress experiments on real radulae [34,35]. Incorporating cyclic loading and wear into future experiments will therefore be essential to evaluate long-term functional performance under biologically realistic feeding regimes, similar to experiments on sea urchin teeth [75].

### 4.6. Collective Effects, Kinematics, and System-Level Implications

The higher stiffness of marginal-teeth variants likely contributed to their superior engagement with substrate and performance, particularly in the stiffer gel. Under natural conditions, closer tooth spacing and coordinated motion would be expected to further amplify these effects via collective interactions between neighboring teeth. Our earlier experiments showed that manipulating spacing alone can strongly alter the efficiency and resistance to failure [47]. Because the present study did not manipulate spacing or include breaking tests, conclusions regarding in vivo failure mechanics remain conservative.

The data from [47] together with the data presented here demonstrate that the collective effect alone does not determine realized performance. Tooth geometry likely defines a functional envelope; the engagement with the substrate is then modulated by kinematics and muscular control in vivo, including the attack angle, stroke amplitude, and timing—which can be highly diverse within Mollusca [14,76,77,78,79,80]. The high sensitivity of central-teeth variants to small geometric changes observed here suggests that these teeth may rely more strongly on precise kinematic tuning, whereas marginal-teeth variants function more robustly across a wider range of operating conditions.

### 4.7. Relation to Other Distributed Mechanical Systems

The radula belongs to a broader class of biological and technical systems in which functional performance emerges from arrays of small distributed interacting elements (distributed mechanical systems, DMSs), rather than from single rigid tools. In such systems, efficiency is governed by the probability of productive engagement, controlled compliance, and collective load sharing, rather than by maximal force generation alone. Comparable principles occur across diverse biological feeding, attachment, and manipulation systems, underscoring the generality of the collective mechanisms identified in such distributed mechanical systems.

Arrays of interacting elements are common in arthropod feeding structures, including serrated mandibles, maxillae, and setal fields, where cutting, scraping, catching, or filtering performance depends on geometry, spacing, and the ability of elements to maintain contact under load [81,82,83,84,85,86,87]. Similarly, crustacean gastric mills consist of coordinated arrays of ossicles and teeth, whose efficiency depends on collective action and contact mechanics in addition to the strength of individual elements [88,89]. In these systems, excessive compliance leads to sliding and energy dissipation, whereas excessive stiffness increases the failure risk—paralleling the balance between deformation, engagement, and efficiency observed in the present study for radular teeth.

Analogous principles also apply in vertebrate dentitions, when considered at the level of functional tooth series rather than individual teeth [90]. In mammalian teeth, effective food processing depends on occlusal geometry, spacing, and coordinated action across multiple elements. As in the present study, increased force is not necessarily translated into increased food breakdown, if the contact geometry promotes compression or sliding rather than penetration [90,91]. This reinforces the interpretation that mechanical work alone is an insufficient predictor of functional yield.

Beyond feeding, strong parallels exist with biological attachment systems such as gecko setae, insect adhesive pads, and spine arrays [92,93,94,95]. These systems rely on large numbers of compliant elements that are individually weak but collectively effective when engagement is reliable. Performance collapses when engagement probability drops below a threshold, even if applied forces increase. This mirrors our observation that collective effects in radular arrays can only enhance performance when individual teeth successfully engage with the substrate.

These comparisons place the radula within a broader framework of DMSs, where performance depends on the interaction between individual-element geometry and array-level coupling, which could also be applied to bioinspired and technical systems, including soft robotic grippers, bristle-based cleaning tools, and abrasive machining [96]. The present results show that tooth shape determines whether productive engagement is possible, while collective interactions determine how forces are distributed and sustained once engagement occurs [47]. This dual dependence explains why marginal teeth—robust, consistently engaging, and less geometry-sensitive—behave as reliable workhorses, whereas central teeth function as more sensitive high-variance elements, whose contribution depends strongly on precise geometry and kinematic context.

### 4.8. Scaling, Material Limits, and Evolutionary Implications

Although the physical models were upscaled (~1:750), the geometric relationships and relative stiffness order were maintained, allowing robust comparative inference. The strong morphological effects observed even in homogeneous materials demonstrate that geometry alone can impose substantial functional constraints.

The pronounced sensitivity of central teeth to small geometric changes suggests that modest phenotypic plasticity could have large functional consequences. In a steep performance landscape, slight developmental adjustments in cusp orientation or stylus geometry may shift teeth between effective engagement and functional failure. This provides a mechanistic framework for previously reported diet- or substrate-induced plasticity in radular morphology, where subtle changes may suffice to tune performance to local conditions [97,98,99,100,101,102,103]. Conversely, the robustness of marginal teeth is consistent with stabilizing selection for reliable performance across a wide range of conditions, supporting the idea that radular evolution balances tunable sensitive elements with robust redundancy-based components.

The high variability and occasional poor performance of central teeth raise the question of why such morphologies persist evolutionarily. Our results suggest that these teeth are not inherently inefficient but conditionally effective: under appropriate geometric alignment and substrate interaction, they can contribute substantially to the material removal. The evolutionary maintenance of such morphologies does not require consistently high performance but rather sufficient payoff under specific conditions to offset periods of low efficiency. Within a system buffered by robust marginal teeth, central teeth may therefore persist as high-variance components that enhance overall feeding capacity without compromising reliability at the system level. Potentially, central teeth may serve a different function than removing food from substrates, e.g., functioning as spacers or joints.

### 4.9. Future Directions

Natural feeding substrates vary in stiffness, abrasiveness, heterogeneity, and microstructural organization. The strong geometry-dependent effects observed here suggest that radular tooth types are functionally differentiated: tuned to distinct microtasks within complex substrates, with robust marginal teeth ensure reliable particle handling, and with more sensitive central teeth contributing under specific geometric and kinematic conditions. Extending this framework to heterogeneous and abrasive substrates will clarify how the mechanical performance relates to ecological specialization.

Together with [47], the present results establish a two-axis framework for radular biomechanics: individual-tooth geometry determines engagement potential, while collective interactions determine how forces are distributed and sustained. Collective effects can only enhance performance if individual teeth reliably engage the substrate; where engagement is geometry-limited, increased work or tighter coupling are not translated into higher yield.

Future studies should explicitly integrate these axes by combining systematic variation in tooth geometry with controlled manipulation of tooth spacing and membrane compliance. Additional priorities include testing more realistic substrates, incorporating cyclic loading to assess fatigue, precisely controlling the attack angle, and developing multi-material or gradient models that better approximate natural stiffness distributions. Ultimately, dynamic radula models [40] integrating compliant membranes and coordinated tooth motion will be essential to bridge static mechanical assays with the emergent feeding behavior of living gastropods, extending the experimental framework established here and in [47]. Additionally, simulations of the biomechanical behavior of radulae including micro-gradients and the physical properties of the food or saliva [104,105] will provide valuable insights into the role of morphology.

## 5. Conclusions

Biomimetic stereolithography-printed radular teeth of *Spekia zonata* revealed pronounced geometry–performance relationships under controlled mechanical conditions. Marginal-tooth variants were mechanically more stable, maintained substrate contact more consistently, and were approximately twice as efficient as central-tooth variants across substrate stiffness levels. In contrast, central-tooth variants exhibited high performance variability, demonstrating that small geometric changes can markedly alter substrate engagement and material removal. Particularly in the stiffer substrate, increased mechanical work did not result in increased yield, highlighting a geometry-limited conversion of work into removal. Together, these findings demonstrate that individual-tooth morphology critically constrains radular efficiency and provide an experimentally grounded framework for disentangling the interacting roles of geometry and collective mechanics in biological feeding interfaces.

## Data Availability

All results can be found in the Supplementary Material. The 3D models are available from WK on request.

## Supplementary Materials

The following supporting information can be downloaded at https://www.mdpi.com/article/10.3390/biomimetics11040246/s1; here, images of the models, the results, and the statistics can be found. **Supplementary Figure S1:** A–B, scanning electron microscopy images of one radula of *Spekia zonata*. Adapted from Krings et al. (2025a) [47]. A, Each tooth row contains on central tooth, one neighbouring lateral tooth and two marginal teeth to each side. C, the 3D model of the radula of *Spekia zonata* which was abstracted to create the models used in this study. Visualized in MeshLab. Adapted from Krings et al. (2025a). **Supplementary Table S1:** Overview about all used tooth model types. Heights and widths of the printed models are given in mm. **Supplementary Figure S2**: All models used in this study, each labelled with model number/stylus type/cusp type. Above, central teeth. Below, marginal teeth. Visualized in MeshLab. **Supplementary Figure S3:** Stylus types of the central tooth. A–B, stylus type I. C–D, stylus type J. E–F, stylus type K. G–H, stylus type L. Left: posterior view. Right: frontal view. Visualized in MeshLab. **Supplementary Figure S4:** Central tooth stylus types side by side. A–C, from left to right: stylus type I, J, K, and P in frontal view. D, stylus types in reversed order, posterior view. Visualized in MeshLab. **Supplementary Figure S5:** Cusp types of the central teeth in different perspectives. A–C, cusp type P. D–F, cusp type N. G–I, cusp type O. J–L, cusp type M. Visualized in MeshLab. **Supplementary Figure 6:** Cusp types of the central teeth side by side. A, dorsal view, from left to right: cusp types O, M, P, N. B–D, models in reversed order, ventral and anterior views. Visualized in MeshLab. **Supplementary Figure S7:** Central tooth models 1–4. Left: anterior view. Right: posterior view. Visualized in MeshLab. **Supplementary Figure S8:** Central tooth models 5–8. Left: anterior view. Right: posterior view. Visualized in MeshLab. **Supplementary Figure S9:** Central tooth models 9–12. Left: anterior view. Right: posterior view. Visualized in MeshLab. **Supplementary Figure S10:** Central tooth models 13–16. Left: anterior view. Right: posterior view. Visualized in MeshLab. **Supplementary Figure S11: **Stylus types of marginal teeth in different perspectives. A–C, stylus type A. D–F, stylus type D. G–I, stylus type C. J–L, stylus type B. Visualized in MeshLab. **Supplementary Figure S12:** Marginal stylus types side by side. A–B, from left to right: stylus types A, B, C, and D, frontal view. C–F, stylus types in reversed order (D, C, B, A), posterior and ventral views. Visualized in MeshLab. **Supplementary Figure S13:** Cusp types of marginal teeth in different perspectives. A–C, cusp type F. D–F, cusp type E. G–I, cusp type G. J–L, cusp type H. Visualized in MeshLab. **Supplementary Figure S14:** Cusp types of marginal teeth in different perspectives. A, C–F, from left to right: cusp types G, H, F, and E, shown from anterior, dorsal, and ventral views. B, models in reversed order from posterior view. Visualized in MeshLab. **Supplementary Figure S15:** Marginal tooth models 1–4. Left: anterior view. Right: posterior view. Visualized in MeshLab. **Supplementary Figure S16:** Marginal tooth models 5–8. Left: anterior view. Right: posterior view. Visualized in MeshLab. **Supplementary Figure S17:** Marginal tooth models 9–12. Left: anterior view. Right: posterior view. Visualized in MeshLab. **Supplementary Figure S18:** Marginal tooth models 13–16. Left: anterior view. Right: posterior view. Visualized in MeshLab. **Supplementary Figure S19:** Print plates of one marginal tooth model. Sixteen teeth of one type are arranged in four rows. Plate width of printed model: 7.4 × 7.4 cm. Visualized in Blender. **Supplementary Table S2:** Stiffness of tooth variants in the bending test. Stiffness is given in N/m. *N* is the sample size used to calculate the mean. Mean stiffness calculated for marginal and central teeth in general are given. **Supplementary Table S3:** Mean values, standard deviation, and maximum values for force, work, and removed gel mass for the empty runs and the individual tooth variants, sorted by model number and gel type. Yellow colour indicates mean values of the respective tooth type, orange colour highlights maximum values within the specific tooth type, and grey highlights minimum values within each tooth type. **Supplementary Table S4: **Removed gel mass, given in g, for each tooth model depending on gel type (0.4% and 0.8%). Percentage of gel-coated (active) teeth are given. **Supplementary Figure S20: **Pull-through test results illustrating the effect of stylus (A) and cusp (B) morphology on feeding performance. For each tooth type, the removed mass of agar gel, given in g, is shown for the soft (0.4%) and stiff (0.8%) substrates, highlighting morphology- and substrate-dependent differences in material removal. **Supplementary Figure S21:** Pull-through test results showing the relationship between removed gel mass, given in g, and mechanical work, given in Nmm, for the different tooth types in the two gels. Data are grouped by stylus types (left) and cusp types (right). **Supplementary Table S5:** Ranking of central tooth efficiency separated by gel type, sorted from least to highest efficiency. Efficiency was calculated as mean work divided by removed mass. Lower values indicate higher efficiency, reflecting a lower energetic cost per unit of food collected. **Supplementary Table S6:** Ranking of marginal tooth efficiency separated by gel type, sorted from least to highest efficiency. Efficiency was calculated as mean work divided by removed mass. Lower values indicate higher efficiency, reflecting a lower energetic cost per unit of food collected. **Supplementary Table S7:** Summed efficiency values of the two gel types, providing an overview of the overall performance of each tooth variant across both substrate conditions. Sorted from least to highest efficiency. Variants with the lowest summed efficiency values performed best on both gel types and therefore rank highest. Efficiency was defined as work per removed mass, such that lower values indicate higher efficiency and higher values indicate less efficient use of work. Summing efficiency values across gel types allows direct comparison among tooth variants and experimental groups. Values were calculated from the individual efficiencies reported in Supplementary Tables S5 and S6.
