# Supplementary material for: Tooth Shape Controls Stiffness and Food Collection Efficiency in Biomimetic Radular Teeth
Source: Biomimetics (Basel). 2026 Apr 3;11(4):246. doi: 10.3390/biomimetics11040246 (PMC13113574; doi:10.3390/biomimetics11040246)
Supplement: Supplementary file 1 [file biomimetics-11-00246-s001.zip › biomimetics-4232328-supplementary.pdf]

Tooth Shape Controls Stiffness and Food Collection Efficiency in Biomimetic Radular Teeth

Wencke Krings [1,2,3,4,*], Tamina Riesel [3], Thomas M. Kaiser [2], Alexander Daasch [2], Ellen Schulz-Kornas [1,2] and Stanislav N. Gorb [4,*]

[1] Department of Cariology, Endodontology and Periodontology, Leipzig University, Liebigstraße 12, 04103 Leipzig, Germany
[2] Section Mammalogy and Palaeoanthropology, Leibniz Institute for the Analysis of Biodiversity Change, Martin-Luther-King-Platz 3, 20146 Hamburg, Germany
[3] Department of Electron Microscopy, Institute of Cell and Systems Biology of Animals, University of Hamburg, Martin-Luther-King-Platz 3, 20146 Hamburg, Germany
[4] Department of Functional Morphology and Biomechanics, Zoological Institute, Kiel University, Am Botanischen Garten 1–9, 24118 Kiel, Germany

* Correspondence: wencke.krings@uni-hamburg.de (W.K.); sgorb@zoologie.uni-kiel.de (S.N.G.)

Supplementary Material

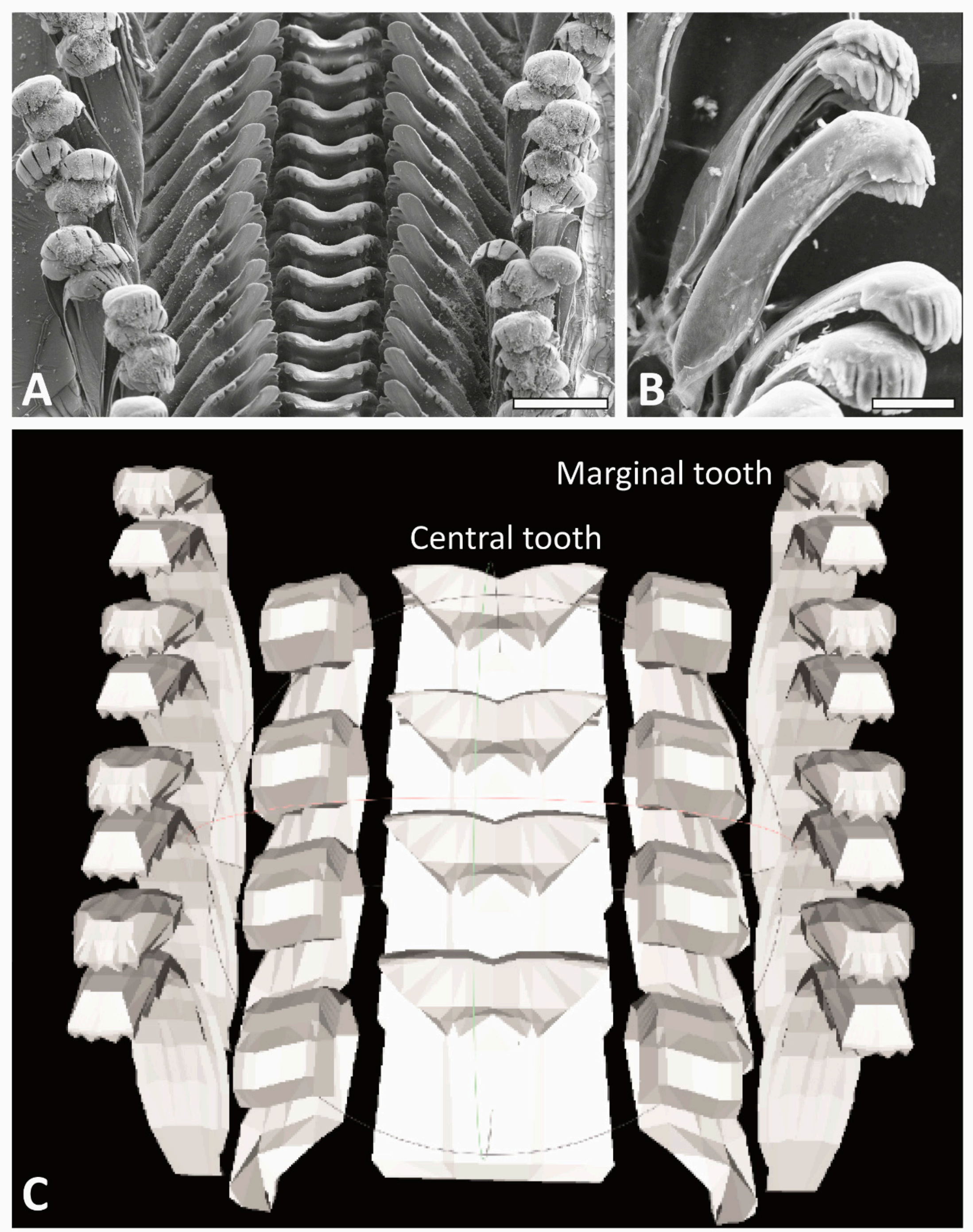

Supplementary Figure S1. A–B, scanning electron microscopy images of one radula of *Spekia zonata*. Adapted from Krings et al. (2025a). A, Each tooth row contains on central tooth, one neighbouring lateral tooth and two marginal teeth to each side. C, the 3D model of the radula of *Spekia zonata* which was abstracted to create the models used in this study. Visualized in MeshLab. Adapted from Krings et al. (2025a).

Supplementary Table S1. Overview about all used tooth model types. Heights and widths of the printed models are given in mm.

Tooth type	Cusp type	Stylus type	Height of tooth	Width of cusp	Model number	see Supplementary Figures
Central	P	I	7.0	12.5	1	2, 7A, 7B
Central	P	J	8.0	12.5	2	2, 7C, 7D
Central	P	K	8.0	12.5	3	2, 7E, 7F
Central	P	L	7.0	12.5	4	2, 7G, 7H
Central	O	I	8.0	12.5	5	2, 8A, 8B
Central	O	J	8.0	12.5	6	2, 8C, 8D
Central	O	K	8.0	12.5	7	2, 8E, 8F
Central	O	L	8.0	12.5	8	2, 8G, 8H
Central	N	I	7.0	12.5	9	2, 9A, 9B
Central	N	J	7.0	12.5	10	2, 9C, 9D
Central	N	K	7.0	12.5	11	2, 9E, 9F
Central	N	L	7.0	12.5	12	2, 9G, 9H
Central	M	I	7.0	12.5	13	2, 10A, 10B
Central	M	J	8.0	12.5	14	2, 10C, 10D
Central	M	K	8.0	12.5	15	2, 10E, 10F
Central	M	L	7.0	12.5	16	2, 10G, 10H
Marginal	E	A	11.0	3.8	1	2, 15A, 15B
Marginal	E	B	12.0	3.8	2	2, 15C, 15D
Marginal	E	C	11.0	4.2	3	2, 15E, 15F
Marginal	E	D	12.0	4.2	4	2, 15G, 15H
Marginal	F	A	12.0	3.8	5	2, 16A, 16B
Marginal	F	B	11.0	3.8	6	2, 16C, 16D
Marginal	F	C	12.0	4.2	7	2, 16E, 16F
Marginal	F	D	12.0	4.2	8	2, 16G, 16H
Marginal	G	A	12.0	4.2	9	2, 17A, 17B
Marginal	G	B	12.0	4.2	10	2, 17C, 17D
Marginal	G	C	12.0	3.8	11	2, 17E, 17F
Marginal	G	D	13.0	3.8	12	2, 17G, 17H
Marginal	H	A	12.0	4.2	13	2, 18A, 18B
Marginal	H	B	12.0	4.2	14	2, 18C, 18D
Marginal	H	C	13.0	3.8	15	2, 18E, 18F
Marginal	H	D	11.0	3.8	16	2, 18G, 18H

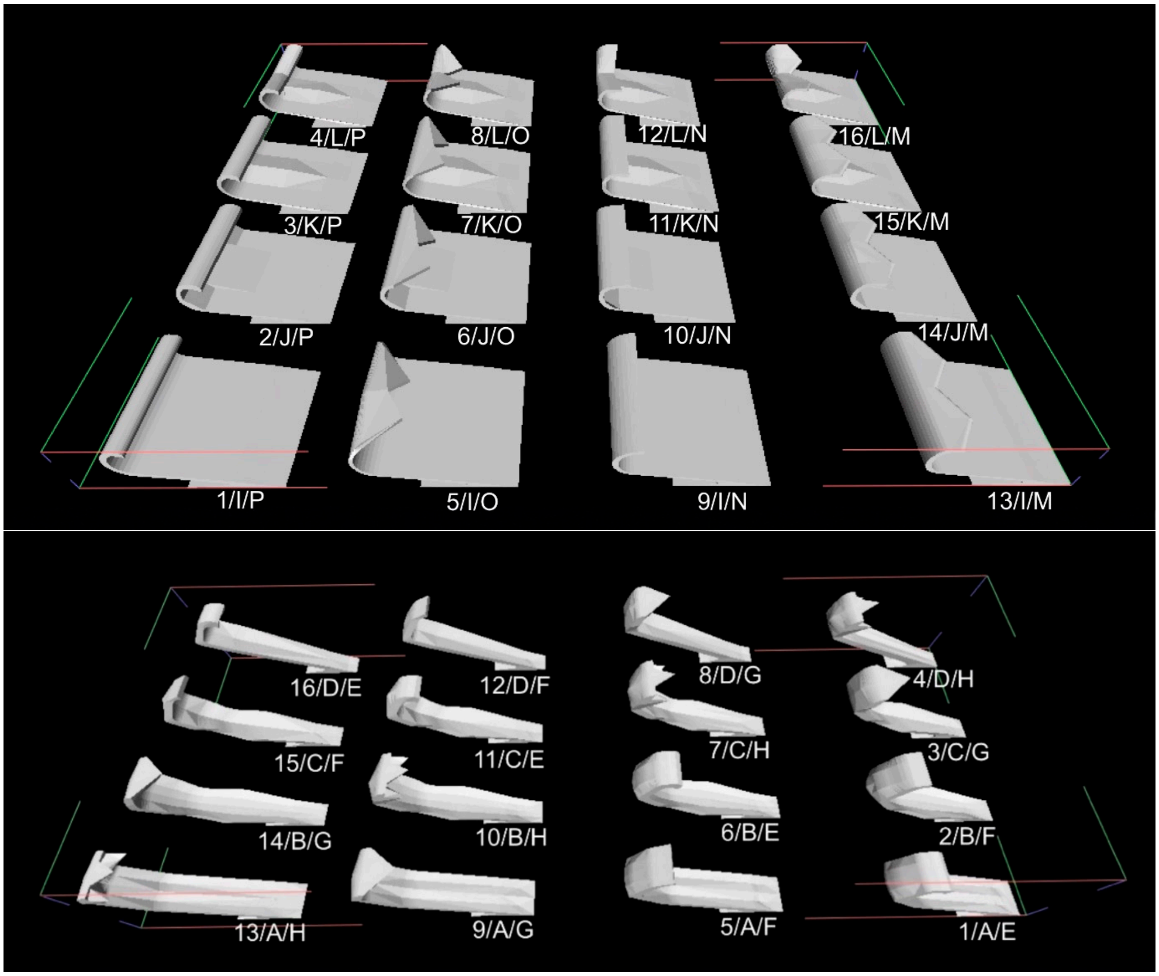

Supplementary Figure S2. All models used in this study, each labelled with model number/stylus type/cusp type. Above, central teeth. Below, marginal teeth. Visualized in MeshLab.

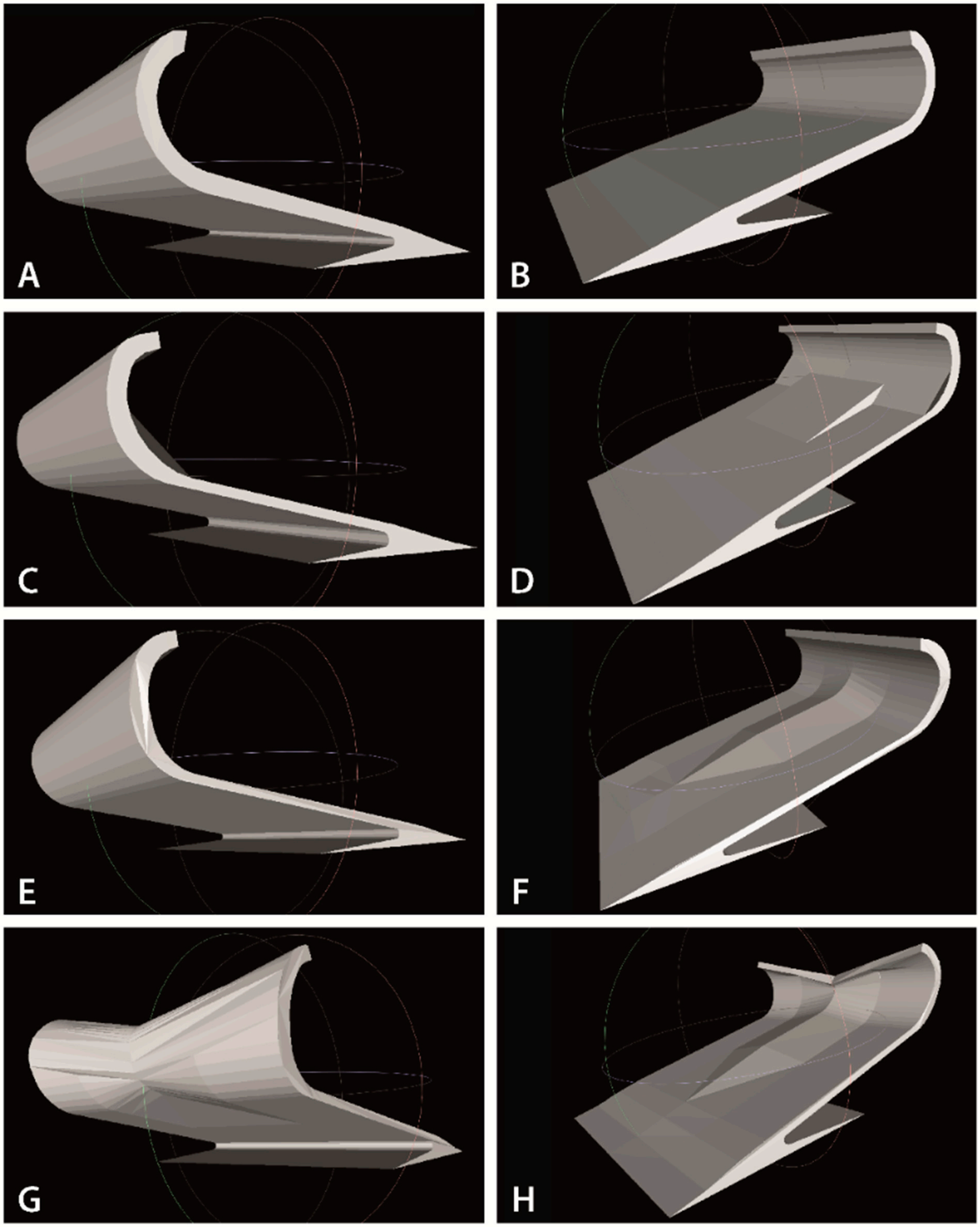

Supplementary Figure S3. Stylus types of the central tooth. A–B, stylus type I. C–D, stylus type J. E–F, stylus type K. G–H, stylus type L. Left: posterior view. Right: frontal view. Visualized in MeshLab.

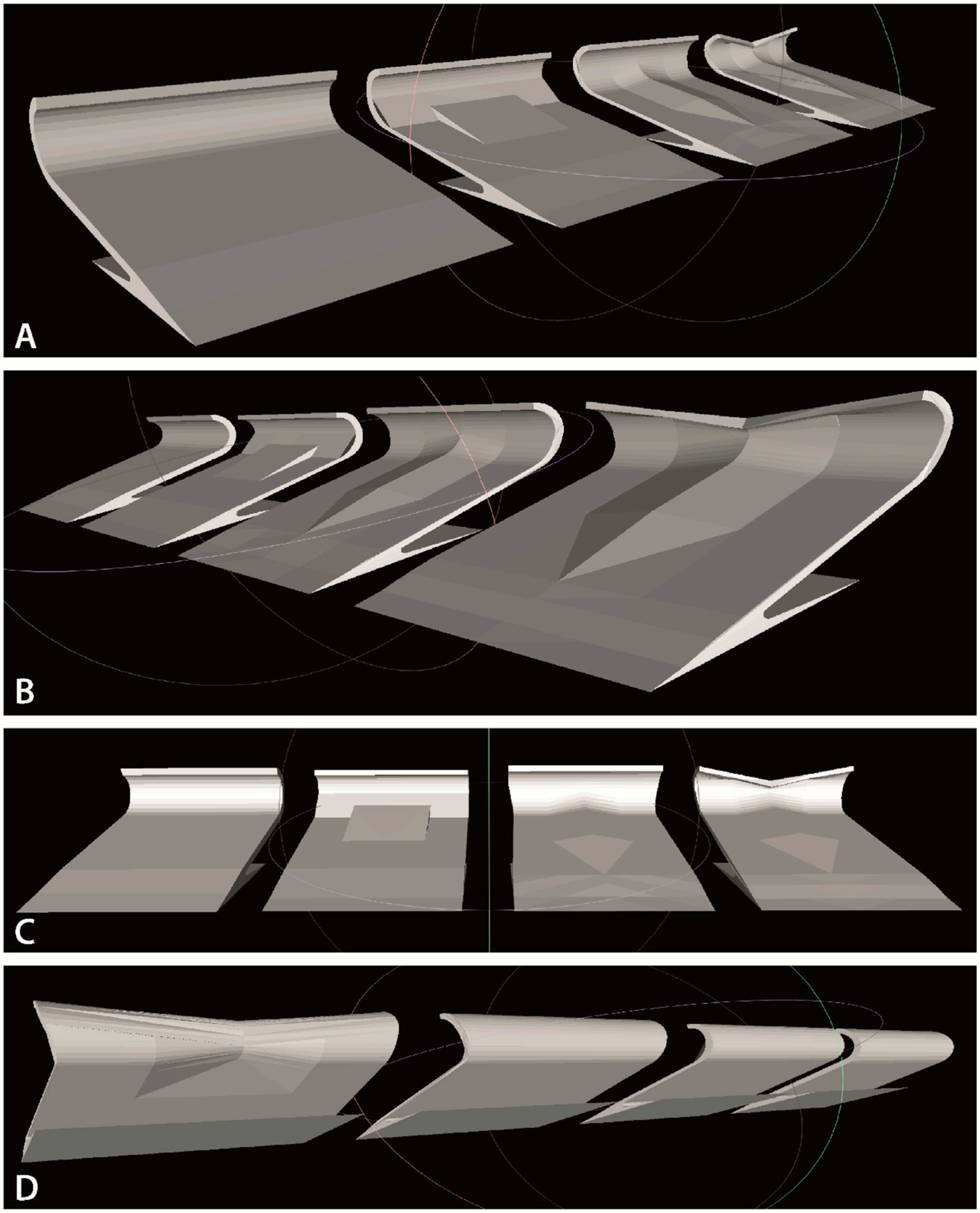

Supplementary Figure S4. Central tooth stylus types side by side. A–C, from left to right: stylus type I, J, K, and P in frontal view. D, stylus types in reversed order, posterior view. Visualized in MeshLab.

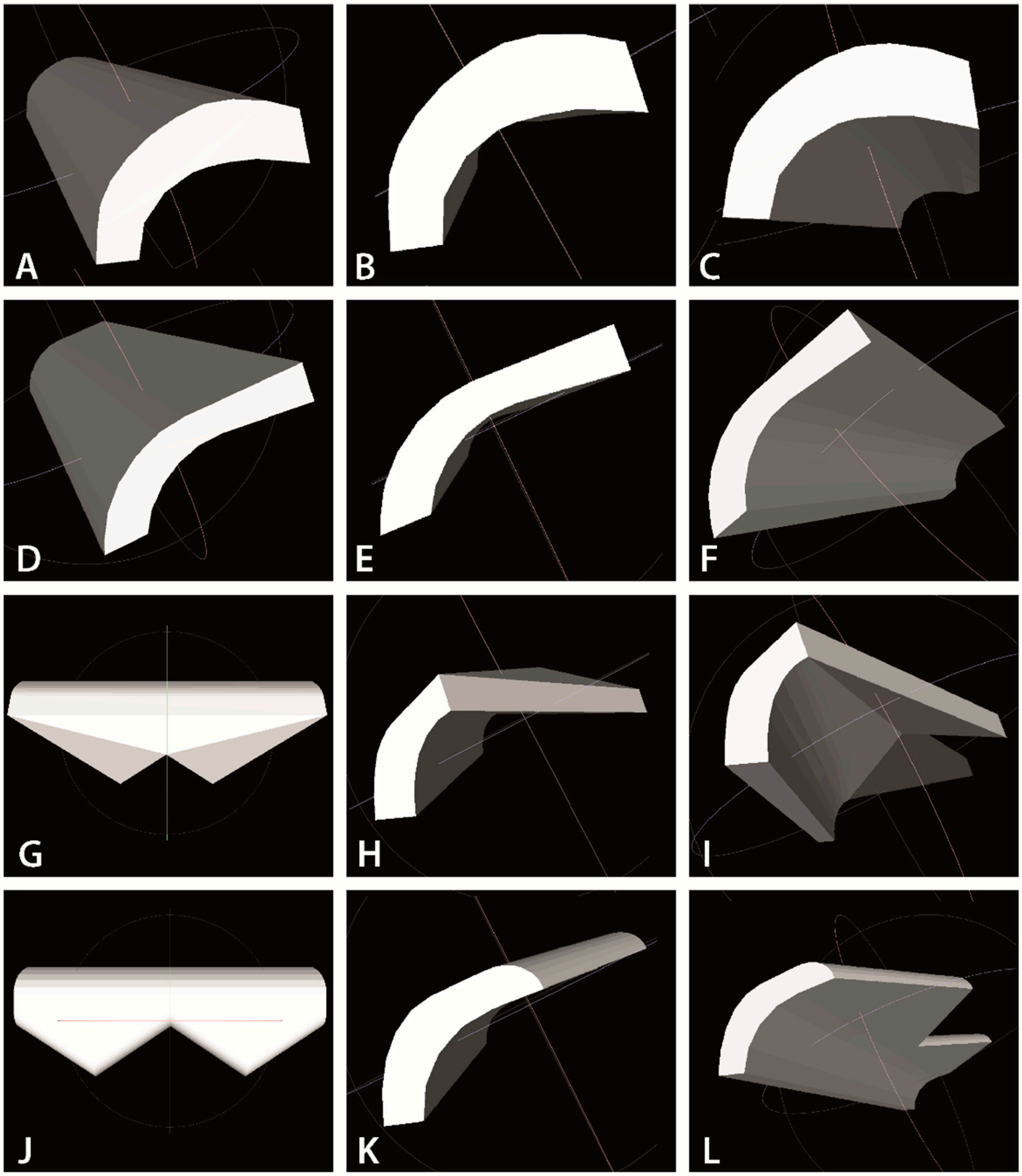

Supplementary Figure S5. Cusp types of the central teeth in different perspectives. A–C, cusp type P. D–F, cusp type N. G–I, cusp type O. J–L, cusp type M. Visualized in MeshLab.

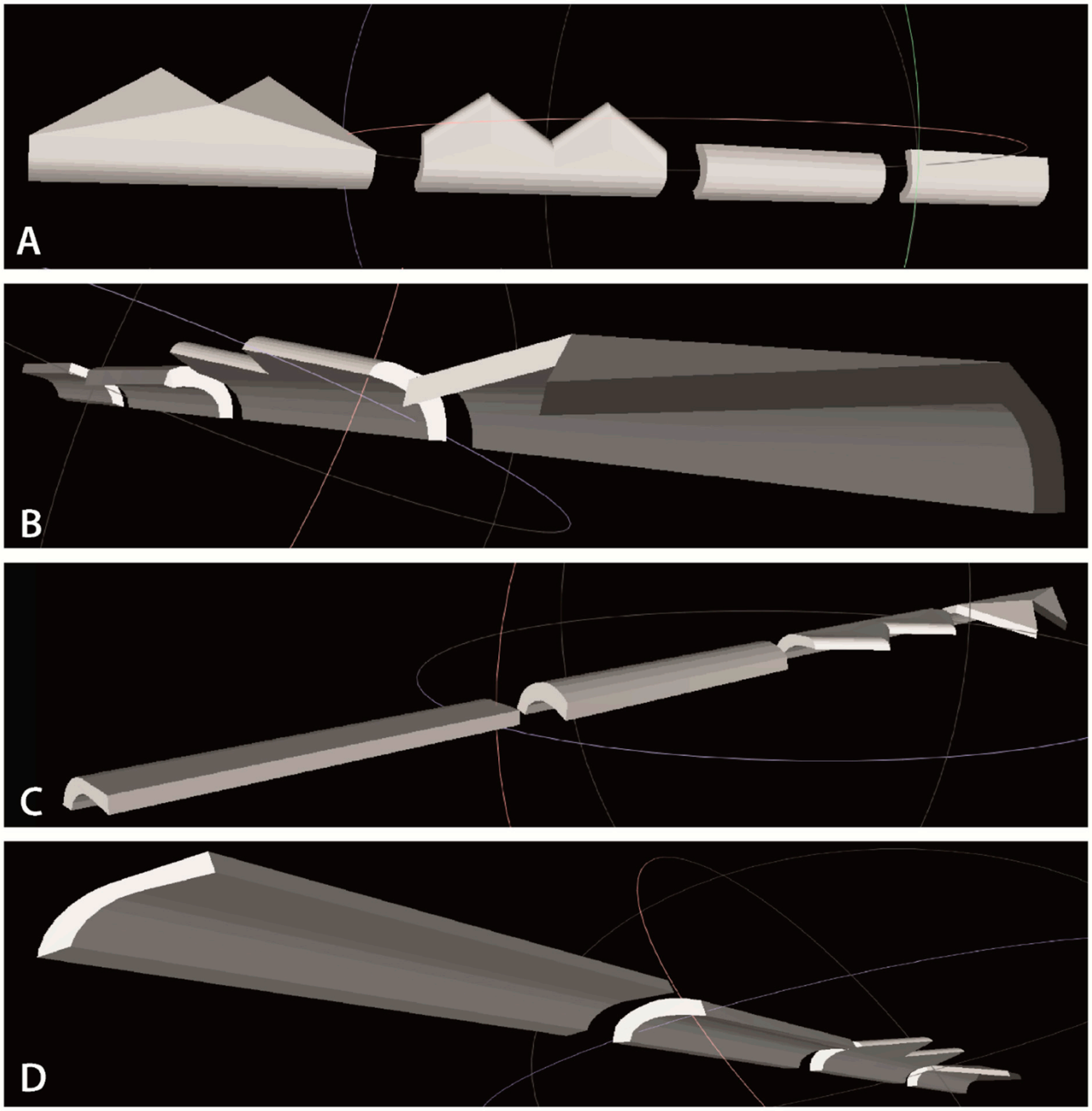

Supplementary Figure S6. Cusp types of the central teeth side by side. A, dorsal view, from left to right: cusp types O, M, P, N. B–D, models in reversed order, ventral and anterior views. Visualized in MeshLab.

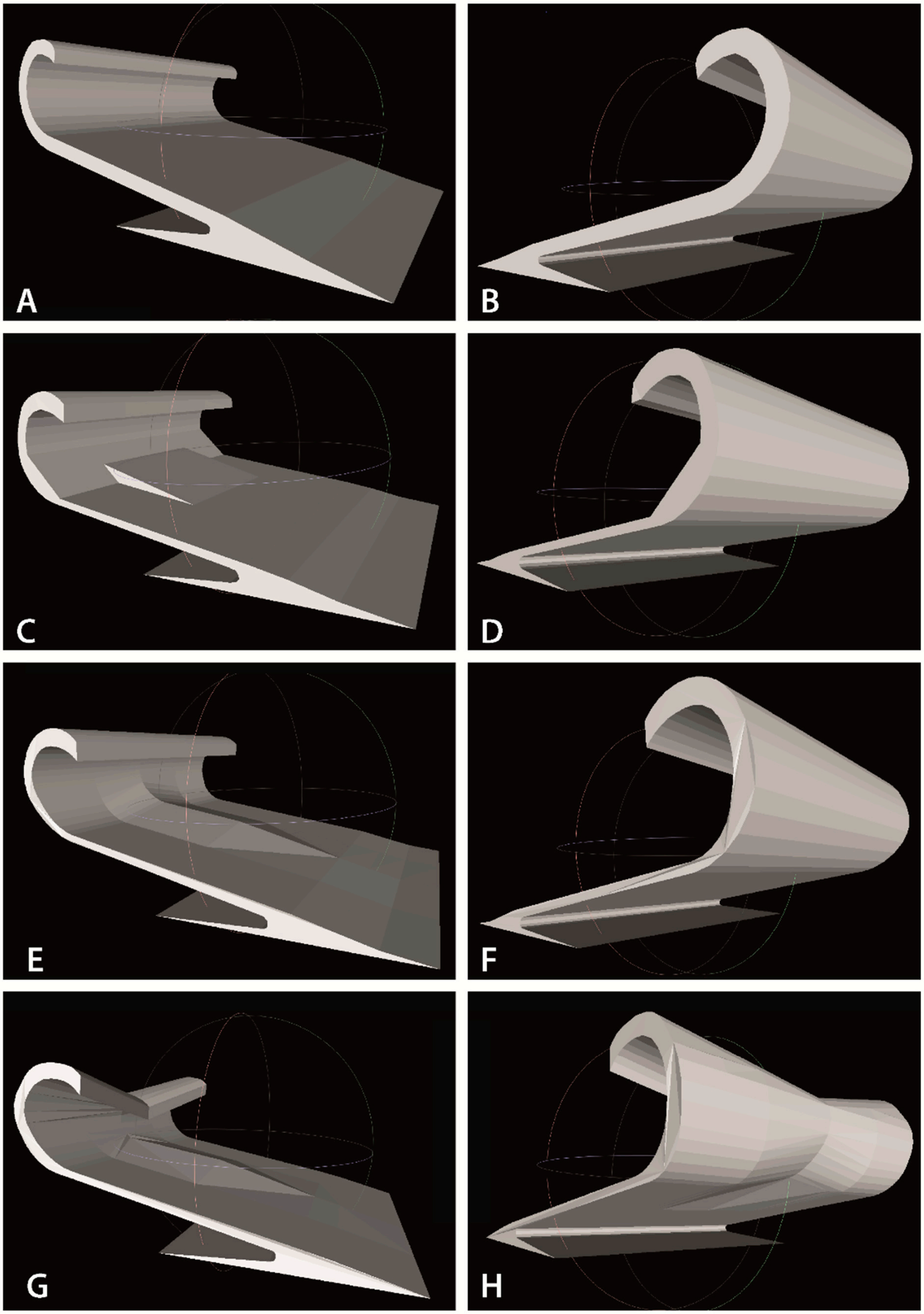

Supplementary Figure S7. Central tooth models 1–4. Left: anterior view. Right: posterior view. Visualized in MeshLab.

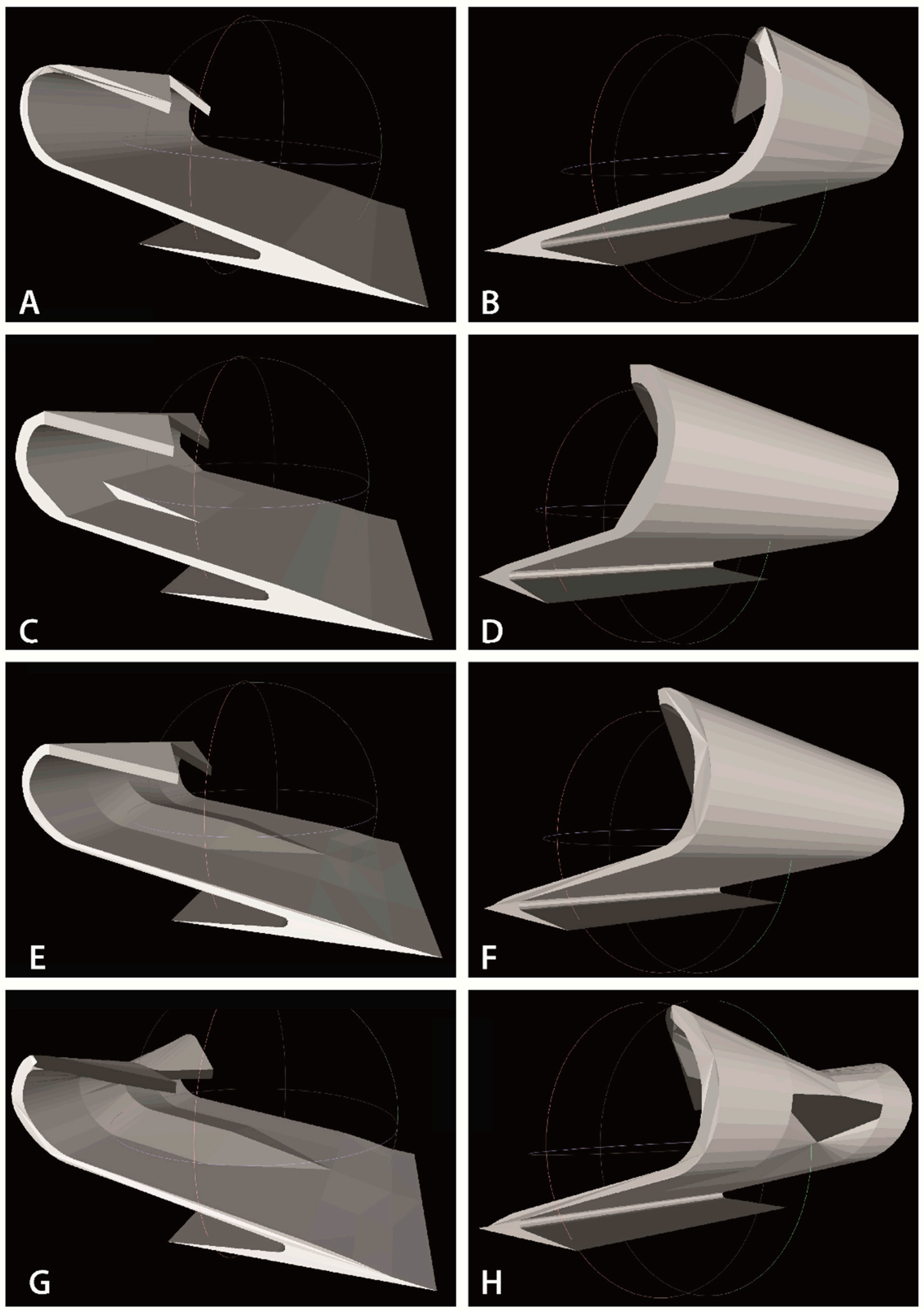

Supplementary Figure S8. Central tooth models 5–8. Left: anterior view. Right: posterior view. Visualized in MeshLab.

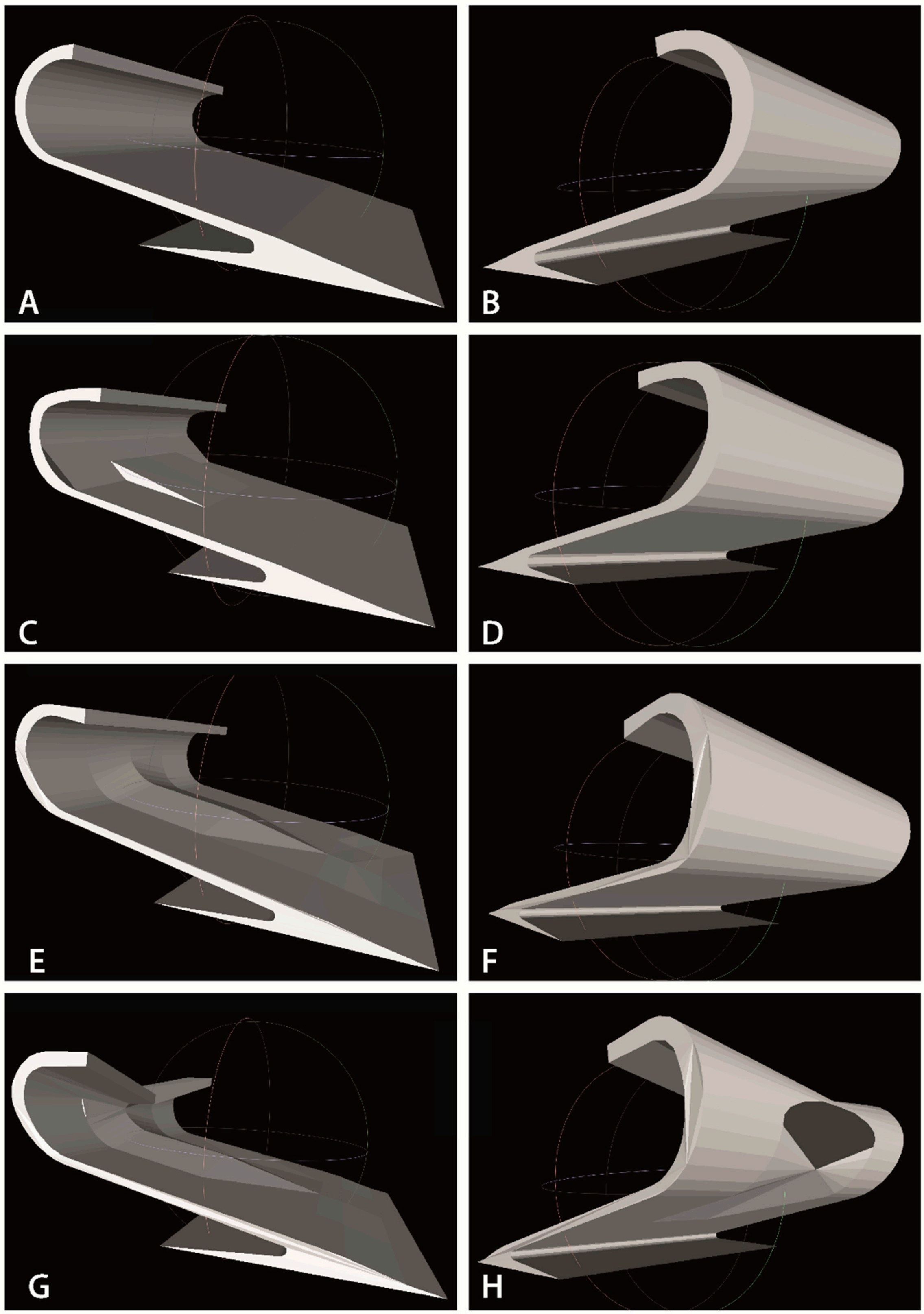

Supplementary Figure S9. Central tooth models 9–12. Left: anterior view. Right: posterior view. Visualized in MeshLab.

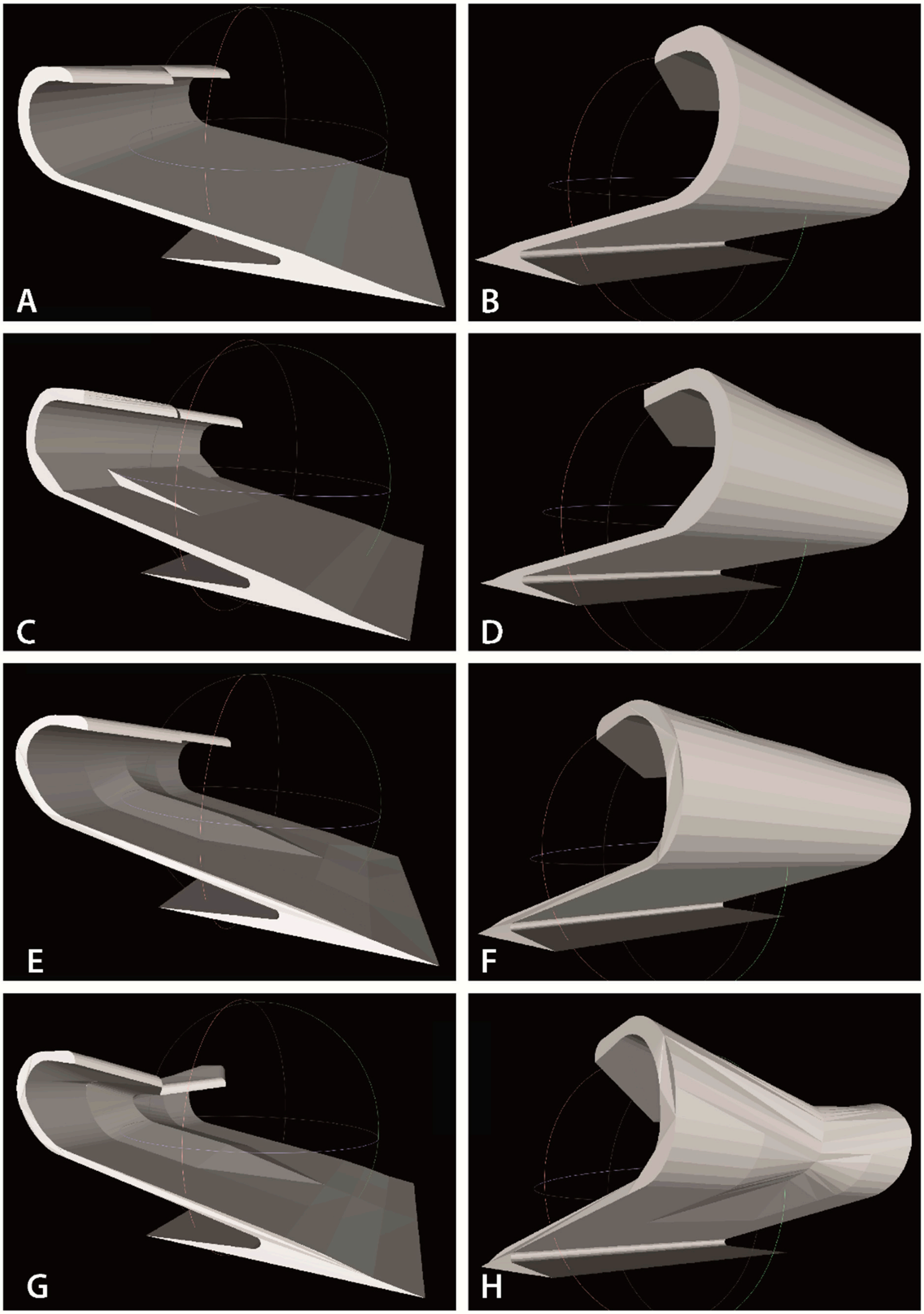

Supplementary Figure S10. Central tooth models 13–16. Left: anterior view. Right: posterior view. Visualized in MeshLab.

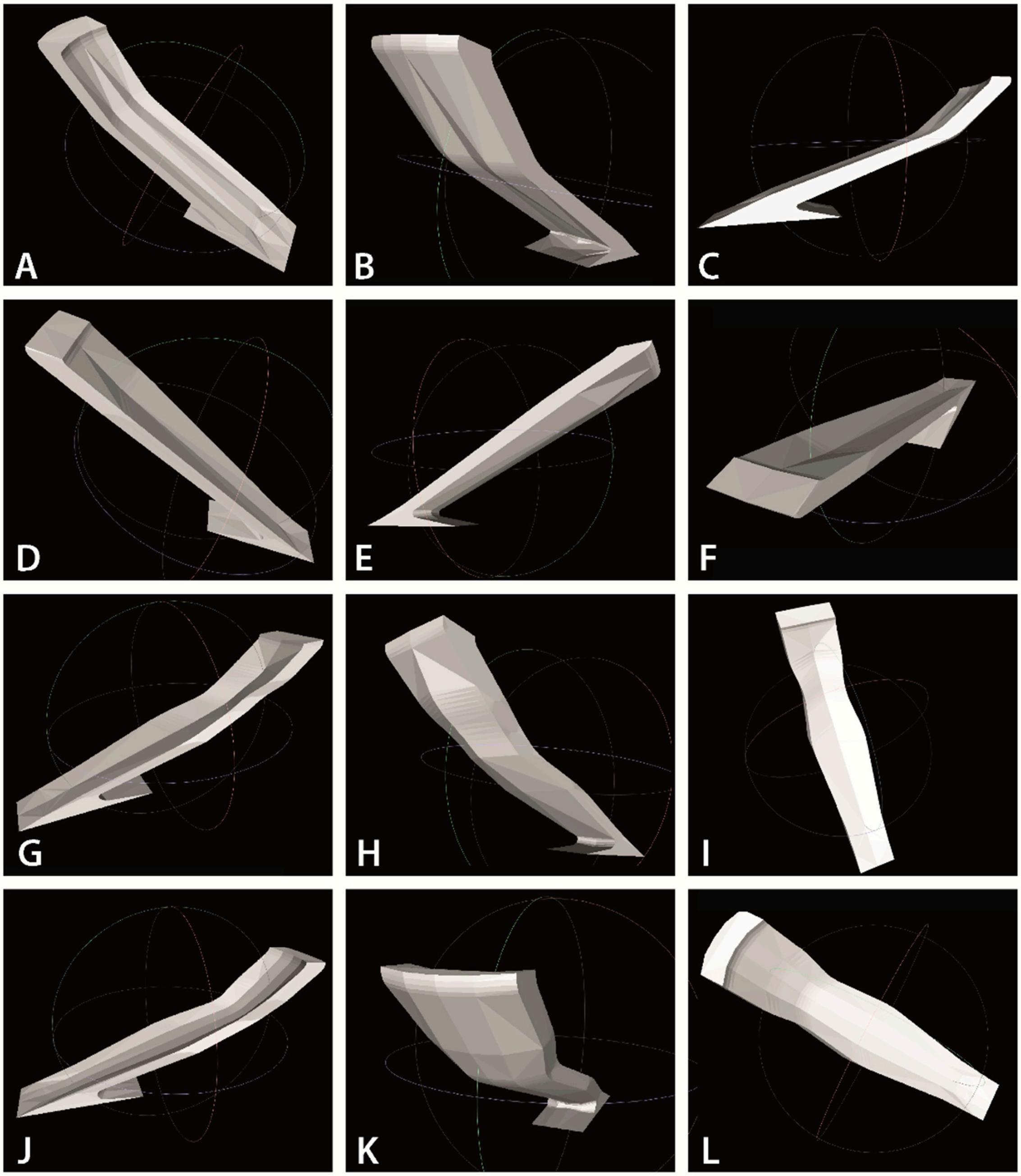

Supplementary Figure S11. Stylus types of marginal teeth in different perspectives. A–C, stylus type A. D–F, stylus type D. G–I, stylus type C. J–L, stylus type B. Visualized in MeshLab.

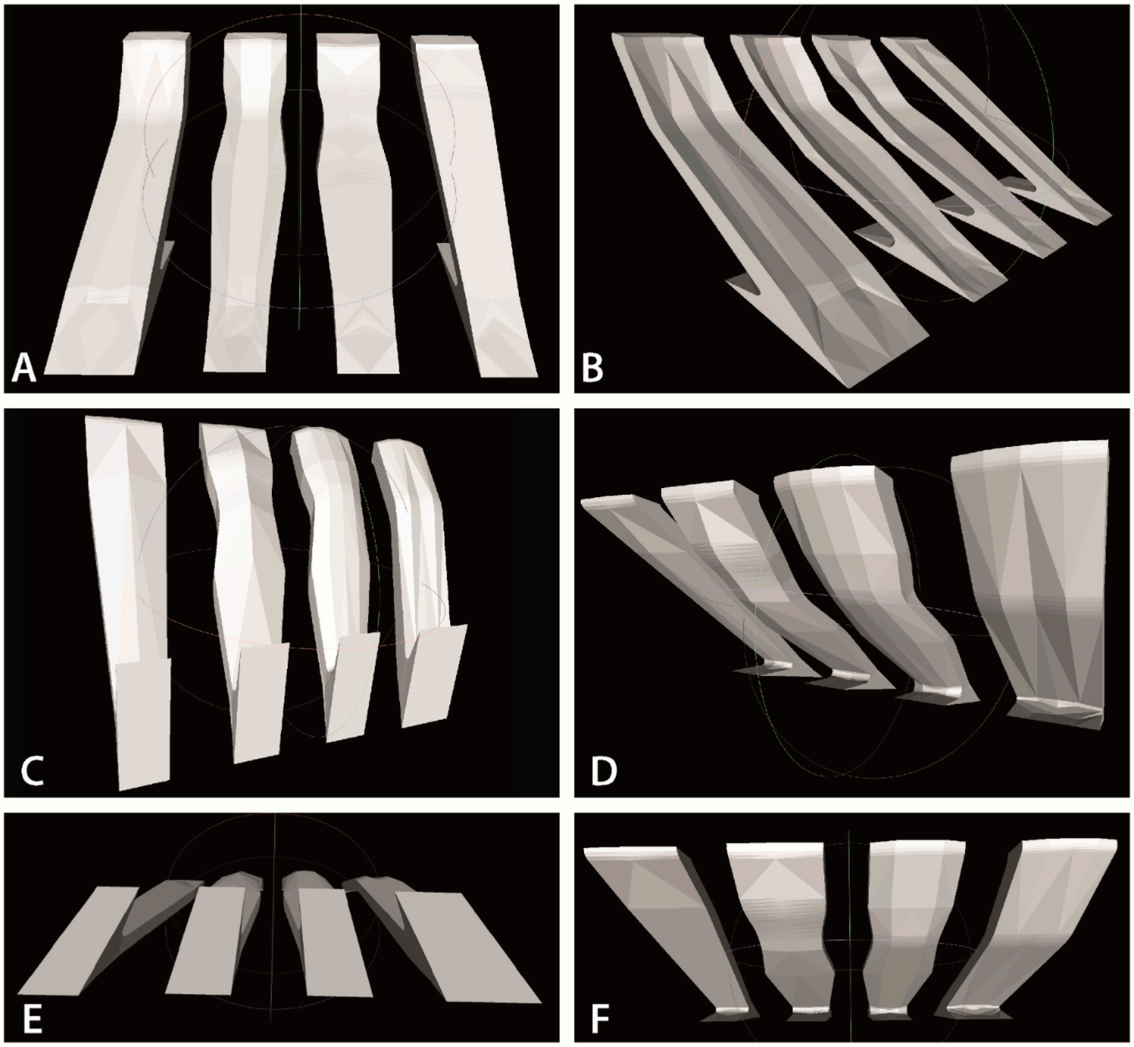

Supplementary Figure S12. Marginal stylus types side by side. A–B, from left to right: stylus types A, B, C, and D, frontal view. C–F, stylus types in reversed order (D, C, B, A), posterior and ventral views. Visualized in MeshLab.

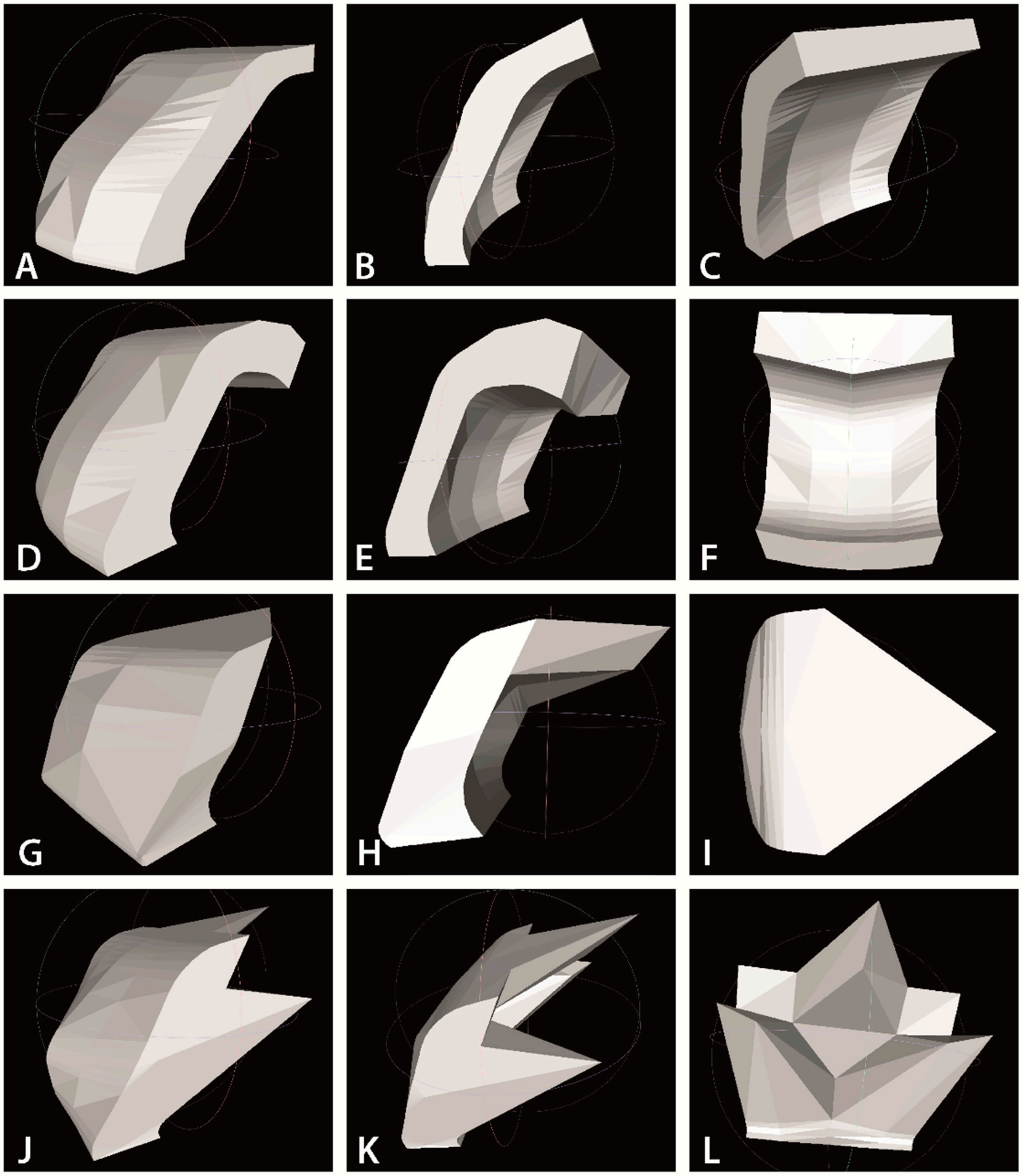

Supplementary Figure S13. Cusp types of marginal teeth in different perspectives. A–C, cusp type F. D–F, cusp type E. G–I, cusp type G. J–L, cusp type H. Visualized in MeshLab.

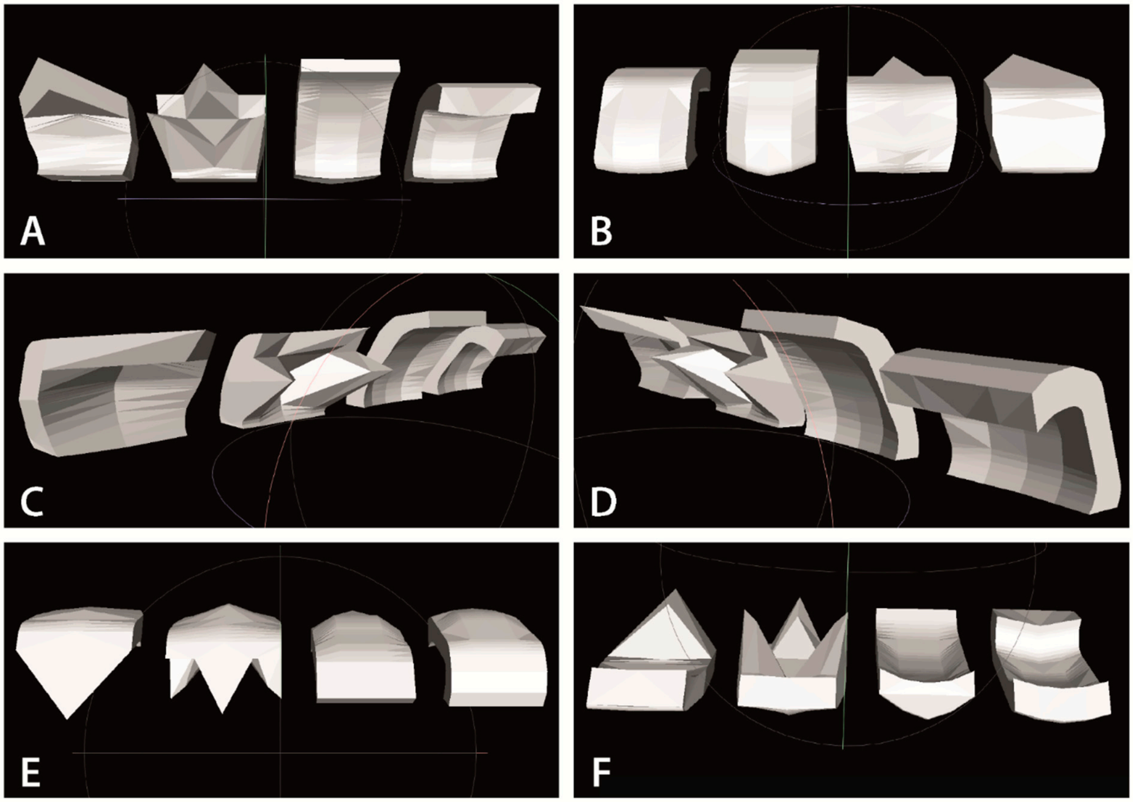

Supplementary Figure S14. Cusp types of marginal teeth in different perspectives. A, C–F, from left to right: cusp types G, H, F, and E, shown from anterior, dorsal, and ventral views. B, models in reversed order from posterior view. Visualized in MeshLab.

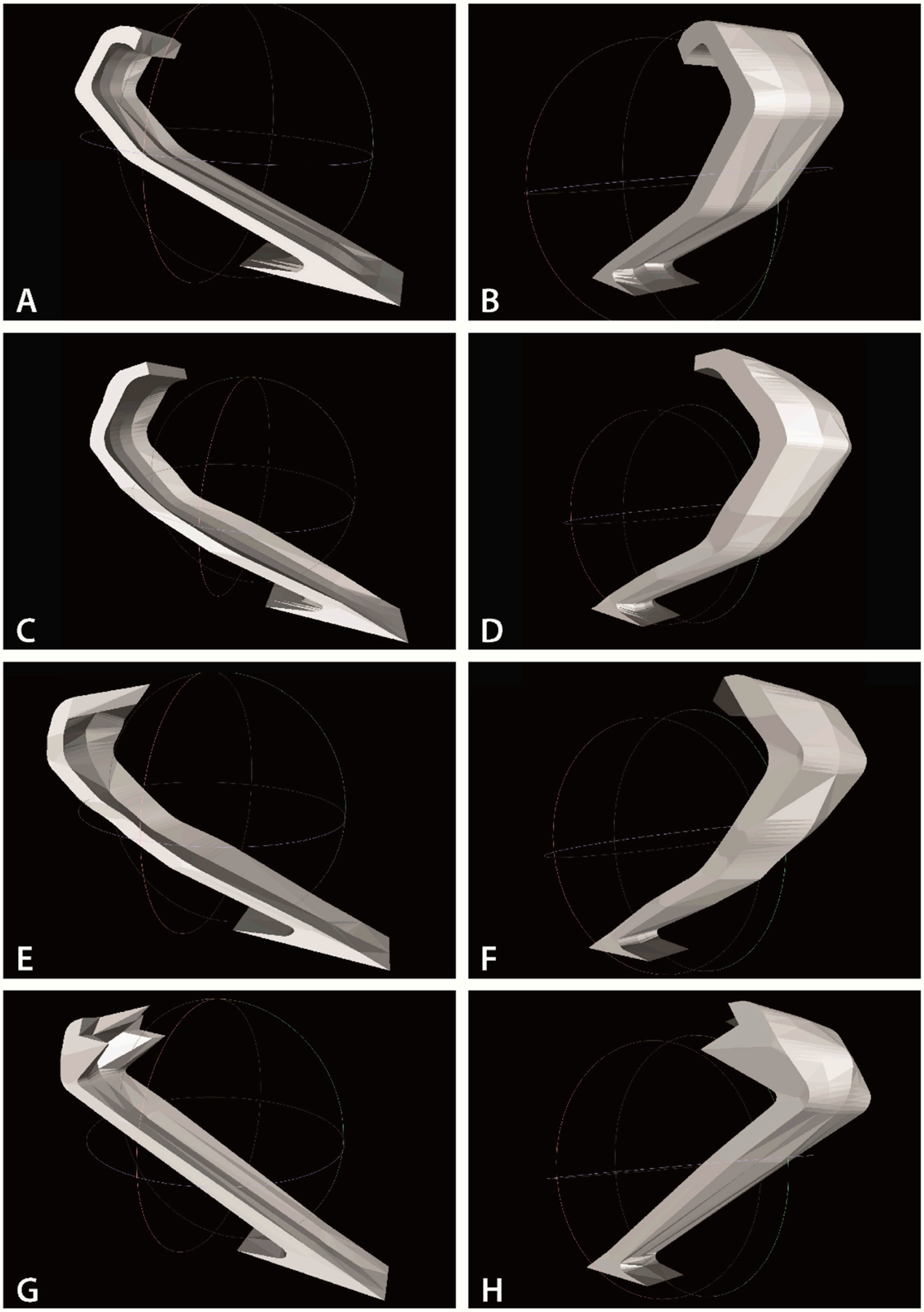

Supplementary Figure S15. Marginal tooth models 1–4. Left: anterior view. Right: posterior view. Visualized in MeshLab.

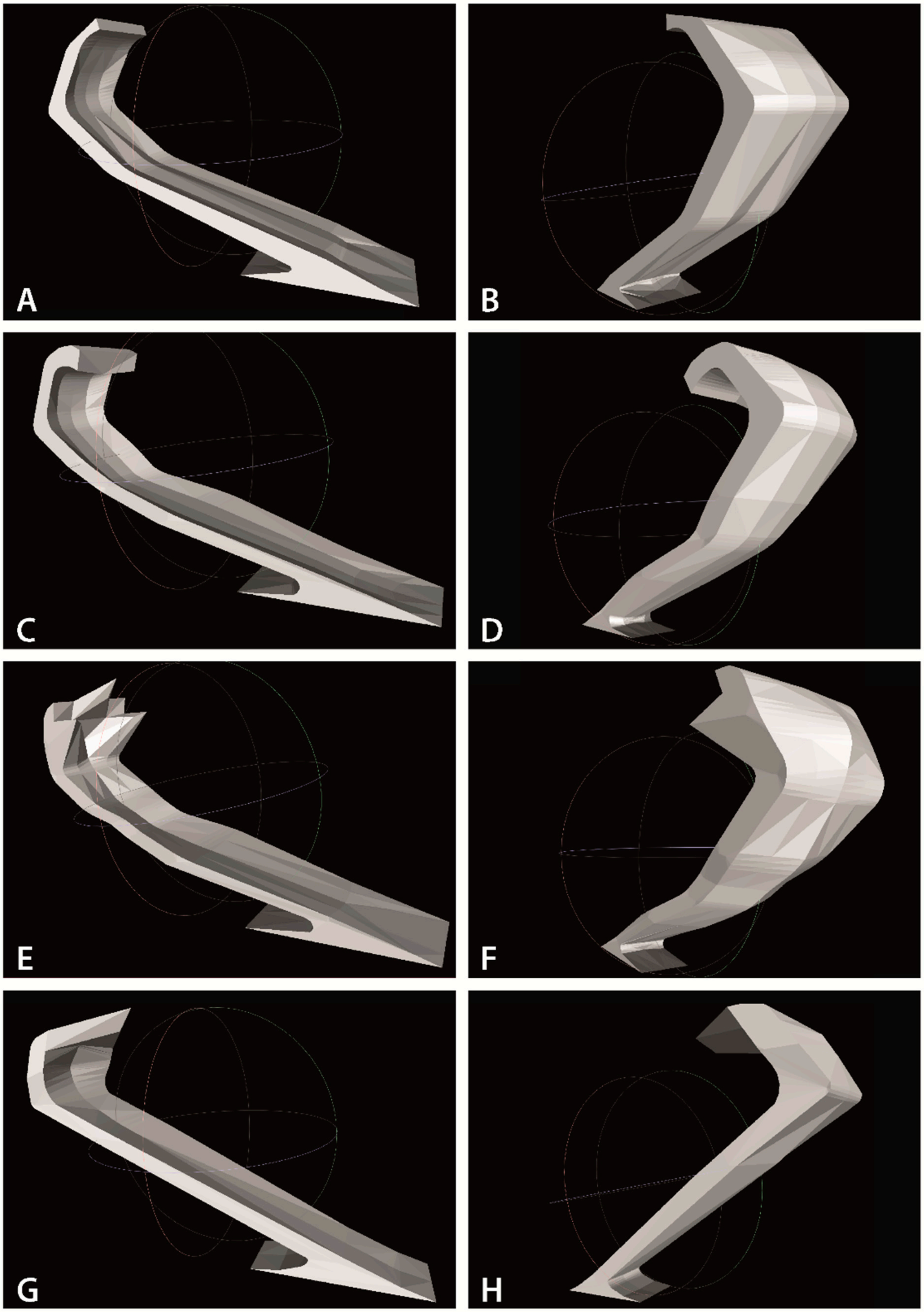

Supplementary Figure S16. Marginal tooth models 5–8. Left: anterior view. Right: posterior view. Visualized in MeshLab.

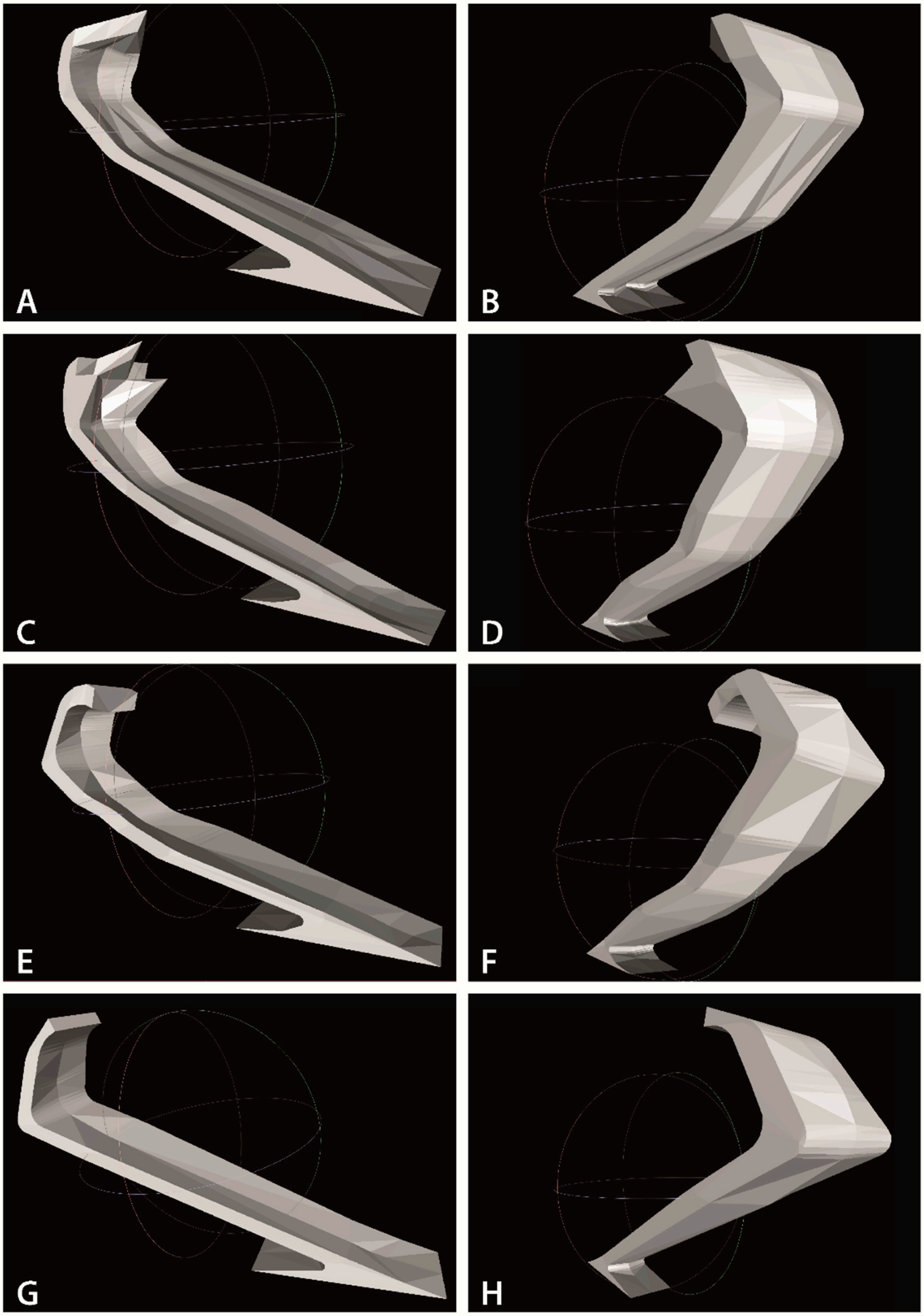

Supplementary Figure S17. Marginal tooth models 9–12. Left: anterior view. Right: posterior view. Visualized in MeshLab.

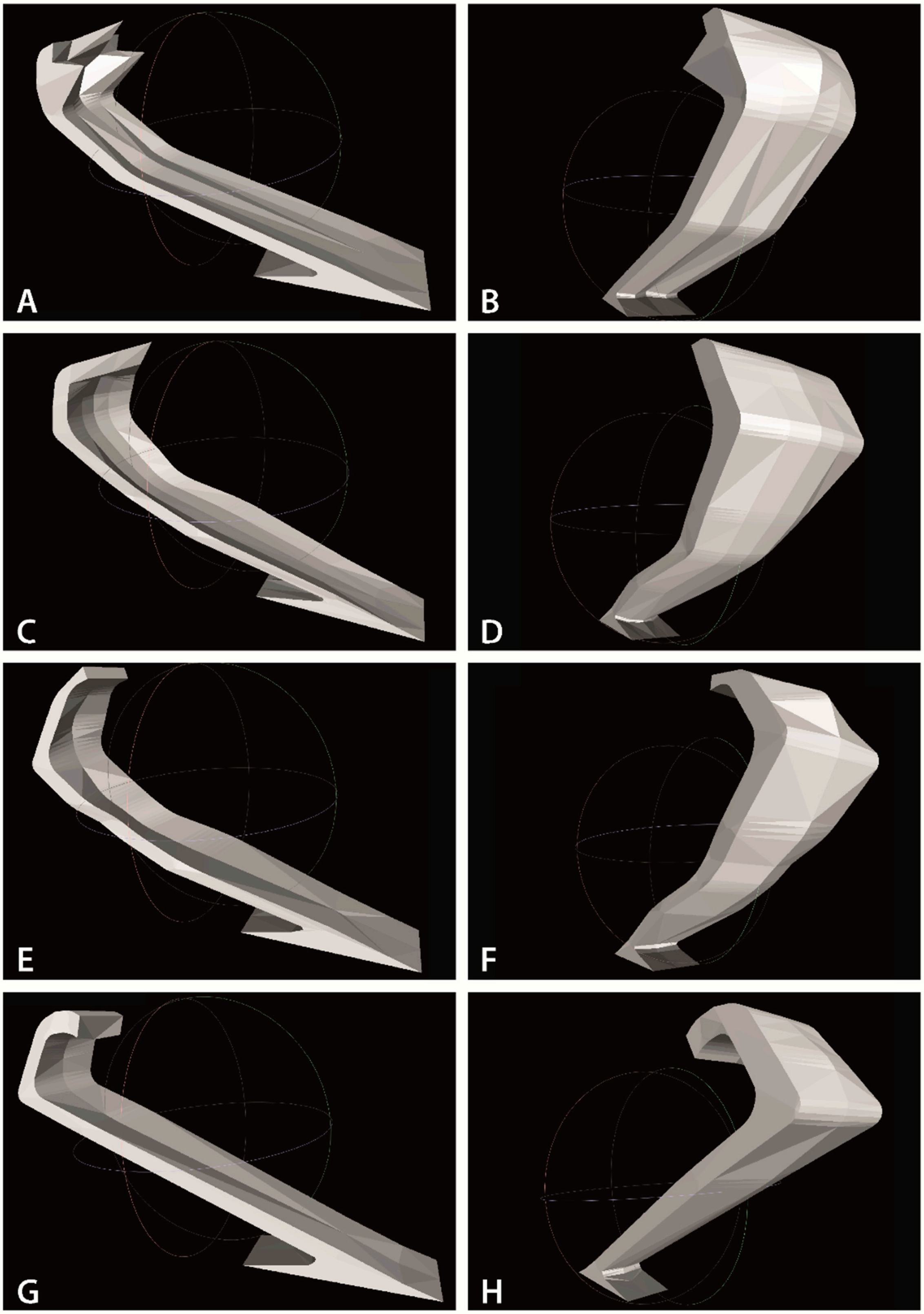

Supplementary Figure S18. Marginal tooth models 13–16. Left: anterior view. Right: posterior view. Visualized in MeshLab.

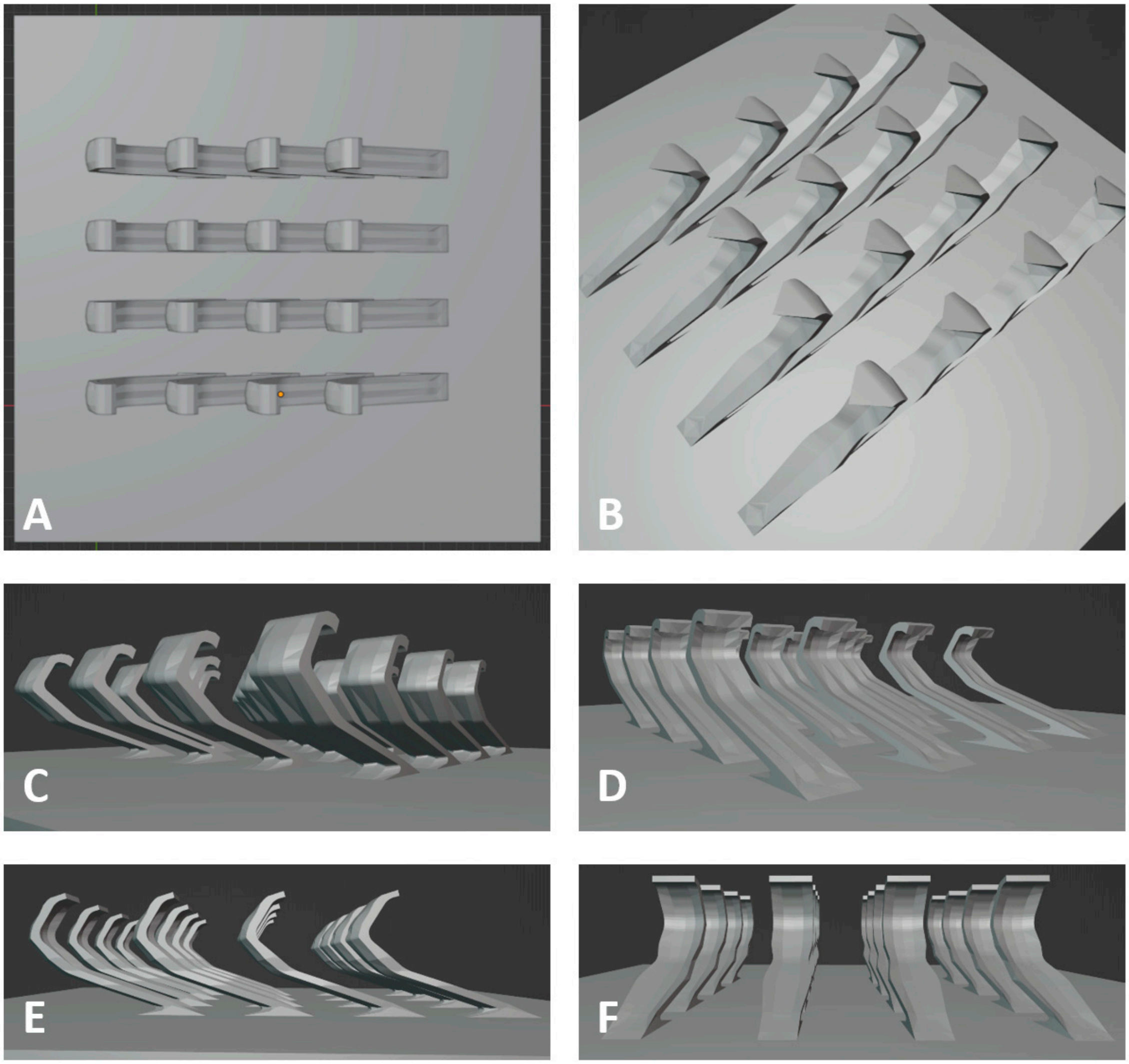

Supplementary Figure S19. Print plates of one marginal tooth model. Sixteen teeth of one type are arranged in four rows. Plate width of printed model: 7.4 × 7.4 cm. Visualized in Blender.

Supplementary Table S2. Stiffness of tooth variants in the bending test. Stiffness is given in N/m. *N* is the sample size used to calculate the mean. Mean stiffness calculated for marginal and central teeth in general are given.

Model number	Central tooth		Marginal tooth	
	Stiffness	*N* of trials	Stiffness	*N* of trials
1	237.19	4	236.43	3
2	181.67	4	768.63	4
3	167.94	4	170.98	4
4	282.50	3	714.42	3
5	653.66	2	606.15	4
6	709.25	4	379.40	4
7	447.49	4	97.76	2
8	370.41	3	118.28	3
9	275.51	3	142.11	4
10	584.90	4	486.65	4
11	505.09	4	118.21	3
12	287.39	3	308.66	4
13	626.00	4	493.89	4
14	279.54	4	2222.22	4
15	432.96	3	252.54	4
16	174.99	4	350.91	4
Mean	388.53	57	466.70	58

Supplementary Table S3. Mean values, standard deviation, and maximum values for force, work, and removed gel mass for the empty runs and the individual tooth variants, sorted by model number and gel type. Yellow colour indicates mean values of the respective tooth type, orange colour highlights maximum values within the specific tooth type, and grey highlights minimum values within each tooth type.

Tooth type	Gel	Model number	Force, N			Work, Nmm			Removed gel, g	
			Mean	SD	Max	Mean	SD	Max	Mean	SD
Marginal	0.4	1	1.46	1.00	3.68	150.91	106.93	306.66	14.40	-
Marginal	0.4	2	1.75	1.27	4.34	178.85	131.88	368.82	12.70	-
Marginal	0.4	3	1.69	1.08	5.83	181.90	120.93	356.30	16.60	-
Marginal	0.4	4	1.63	1.17	3.88	174.15	122.51	343.42	11.20	-
Marginal	0.4	5	1.80	1.24	3.86	195.13	133.61	379.51	14.90	-
Marginal	0.4	6	3.39	2.50	4.21	179.57	126.11	367.51	27.90	-
Marginal	0.4	7	1.97	1.20	5.71	214.64	140.22	414.61	21.10	-
Marginal	0.4	8	1.51	1.11	3.92	158.71	114.57	319.35	11.40	-
Marginal	0.4	9	1.17	0.82	3.38	124.66	86.57	247.03	24.60	-
Marginal	0.4	10	1.66	1.05	3.90	183.58	119.80	350.06	18.50	-
Marginal	0.4	11	1.46	0.88	4.23	161.04	103.39	308.64	13.50	-
Marginal	0.4	12	1.51	0.90	3.91	167.55	106.58	317.61	17.00	-
Marginal	0.4	13	1.53	1.01	5.28	171.85	110.41	322.33	26.60	-
Marginal	0.4	14	1.54	1.05	4.64	166.81	113.93	326.03	21.50	-
Marginal	0.4	15	1.57	1.02	4.14	169.38	113.75	332.37	23.00	-
Marginal	0.4	16	1.34	0.91	3.83	140.24	97.14	282.05	13.90	-
Marginal	0.4		1.58	1.08	5.83	169.94	114.88	414.61	18.05	4.80
Marginal	0.8	1	3.23	2.55	7.69	334.65	248.97	680.55	20.90	-
Marginal	0.8	2	3.84	3.04	8.79	385.90	296.48	810.14	19.80	-
Marginal	0.8	3	2.94	2.33	7.28	301.61	228.76	619.95	19.30	-
Marginal	0.8	4	3.72	2.73	8.50	384.47	281.88	784.50	14.60	-
Marginal	0.8	5	3.30	2.45	7.23	353.01	249.98	696.48	19.80	-
Marginal	0.8	6	1.74	1.15	8.23	369.55	253.69	714.18	24.60	-
Marginal	0.8	7	1.75	1.14	4.20	183.40	123.14	368.18	9.90	-
Marginal	0.8	8	2.21	1.43	4.46	239.10	159.75	467.07	15.30	-
Marginal	0.8	9	3.06	2.30	7.27	323.15	233.71	645.59	17.80	-
Marginal	0.8	10	3.34	2.48	7.72	348.71	252.56	704.12	16.70	-
Marginal	0.8	11	2.62	1.96	6.52	260.74	193.52	552.55	18.10	-
Marginal	0.8	12	3.50	2.65	8.54	374.69	266.86	737.76	21.10	-
Marginal	0.8	13	2.59	1.73	7.48	283.63	188.52	545.72	22.20	-

Marginal	0.8	14	3.36	2.86	8.12	348.64	269.67	707.79	19.80	-
Marginal	0.8	15	3.51	2.68	7.81	369.88	270.59	740.79	19.20	-
Marginal	0.8	16	3.23	2.30	7.04	337.06	241.71	681.86	10.20	-
Marginal	0.8		3.10	2.44	8.79	324.89	239.18	810.14	18.08	4.68
Central	0.4	1	1.21	0.70	4.57	120.99	80.17	254.96	2.40	-
Central	0.4	2	1.17	0.65	3.73	123.93	76.15	246.90	4.10	-
Central	0.4	3	1.51	0.97	4.92	159.99	108.34	319.39	11.30	-
Central	0.4	4	0.95	0.62	2.97	97.38	66.73	199.71	5.90	-
Central	0.4	5	0.94	0.28	2.45	111.27	52.66	197.40	2.10	-
Central	0.4	6	0.65	0.39	1.83	68.00	42.64	136.50	2.90	-
Central	0.4	7	1.49	0.93	3.74	144.37	103.61	314.94	1.90	-
Central	0.4	8	1.46	1.14	3.99	149.39	110.45	309.04	23.60	-
Central	0.4	9	1.36	0.77	3.63	158.23	92.90	288.04	13.40	-
Central	0.4	10	1.45	1.00	4.26	159.00	107.10	305.94	20.90	-
Central	0.4	11	1.96	1.28	5.40	215.01	140.39	414.64	28.40	-
Central	0.4	12	1.66	1.25	4.08	178.94	125.60	351.03	20.20	-
Central	0.4	13	1.16	0.74	2.87	125.91	81.57	244.19	15.30	-
Central	0.4	14	2.04	1.44	5.35	216.74	148.42	429.91	24.00	-
Central	0.4	15	2.36	1.74	6.21	248.65	178.77	497.55	29.30	-
Central	0.4	16	1.27	0.90	3.45	124.31	92.98	268.21	9.80	-
Central	0.4		1.41	1.08	6.21	150.13	112.97	497.55	13.47	9.84
Central	0.8	1	1.56	1.18	5.19	169.64	108.77	327.86	1.80	-
Central	0.8	2	2.39	1.87	9.26	255.52	167.85	504.24	2.90	-
Central	0.8	3	1.67	1.25	6.31	191.65	110.69	352.49	3.00	-
Central	0.8	4	1.13	0.90	5.11	127.65	71.96	237.43	2.50	-
Central	0.8	5	0.94	0.63	3.80	108.50	61.00	197.59	3.40	-
Central	0.8	6	1.06	0.57	2.69	125.27	68.60	223.82	2.90	-
Central	0.8	7	3.99	2.81	8.75	404.22	294.54	842.54	6.10	-
Central	0.8	8	2.09	1.67	8.66	237.57	141.59	440.19	5.90	-
Central	0.8	9	3.67	2.69	10.24	409.18	260.20	775.28	14.40	-
Central	0.8	10	4.33	3.21	12.22	451.06	322.35	911.55	12.30	-
Central	0.8	11	3.49	3.02	12.28	388.30	264.74	735.88	11.00	-
Central	0.8	12	2.78	2.09	8.75	289.47	193.22	587.05	13.70	-
Central	0.8	13	2.84	2.58	10.41	301.95	222.60	601.01	15.70	-
Central	0.8	14	3.95	3.20	12.30	421.17	300.86	833.82	13.60	-
Central	0.8	15	3.18	2.50	8.85	329.65	239.75	670.24	16.30	-
Central	0.8	16	3.34	2.64	10.47	362.82	254.43	706.12	9.10	-
Central	0.8		2.65	2.49	12.30	285.85	234.06	911.55	8.41	5.59
Empty run		1	0.37	0.30	2.86	39.33	20.37	77.69	0.00	-
Empty run		2	0.16	0.22	1.72	14.11	9.69	34.64	0.00	-
Empty run		3	0.51	0.25	2.79	57.77	27.20	108.11	0.00	-
Empty run		4	0.58	0.23	2.35	66.99	30.32	121.51	0.00	-
Empty run		5	0.59	0.26	3.16	67.73	31.09	124.70	0.00	-
Empty run			0.44	0.30	3.16	49.19	31.85	124.70	0.00	-

Supplementary Table S4. Removed gel mass, given in g, for each tooth model depending on gel type (0.4% and 0.8%). Percentage of gel-coated (active) teeth are given.

Tooth	Central tooth				Marginal tooth			
Gel	0.4 %		0.8 %		0.4 %		0.8 %	
Model number	Removed gel	Percentage of active teeth	Removed gel	Percentage of active teeth	Removed gel	Percentage of active teeth	Removed gel	Percentage of active teeth
1	2.4	56.3	1.8	56.3	14.4	100.0	20.9	100.0
2	4.1	62.5	2.9	25.0	12.7	92.3	19.8	92.3
3	11.3	93.8	3.0	25.0	16.6	100.0	19.3	100.0
4	5.9	100.0	2.5	37.5	11.2	100.0	14.6	100.0
5	2.1	43.8	3.4	100.0	14.9	100.0	19.8	93.8
6	2.9	50.0	2.9	37.5	27.9	100.0	24.6	100.0
7	1.9	56.3	6.1	87.5	21.1	100.0	9.9	100.0
8	23.6	93.8	5.9	87.5	11.4	100.0	15.3	100.0
9	13.4	93.8	14.4	68.8	24.6	100.0	17.8	100.0
10	20.9	81.3	12.3	93.8	18.5	100.0	16.7	100.0
11	28.4	100.0	11.0	100.0	13.5	100.0	18.1	100.0
12	20.2	100.0	13.7	81.3	17.0	100.0	21.1	93.8
13	15.3	81.3	15.7	87.5	26.6	100.0	22.2	93.8
14	24.0	100.0	13.6	100.0	21.5	100.0	19.8	100.0
15	29.3	100.0	16.3	100.0	23.0	100.0	19.2	100.0
16	9.8	93.8	9.1	93.8	13.9	100.0	10.2	100.0

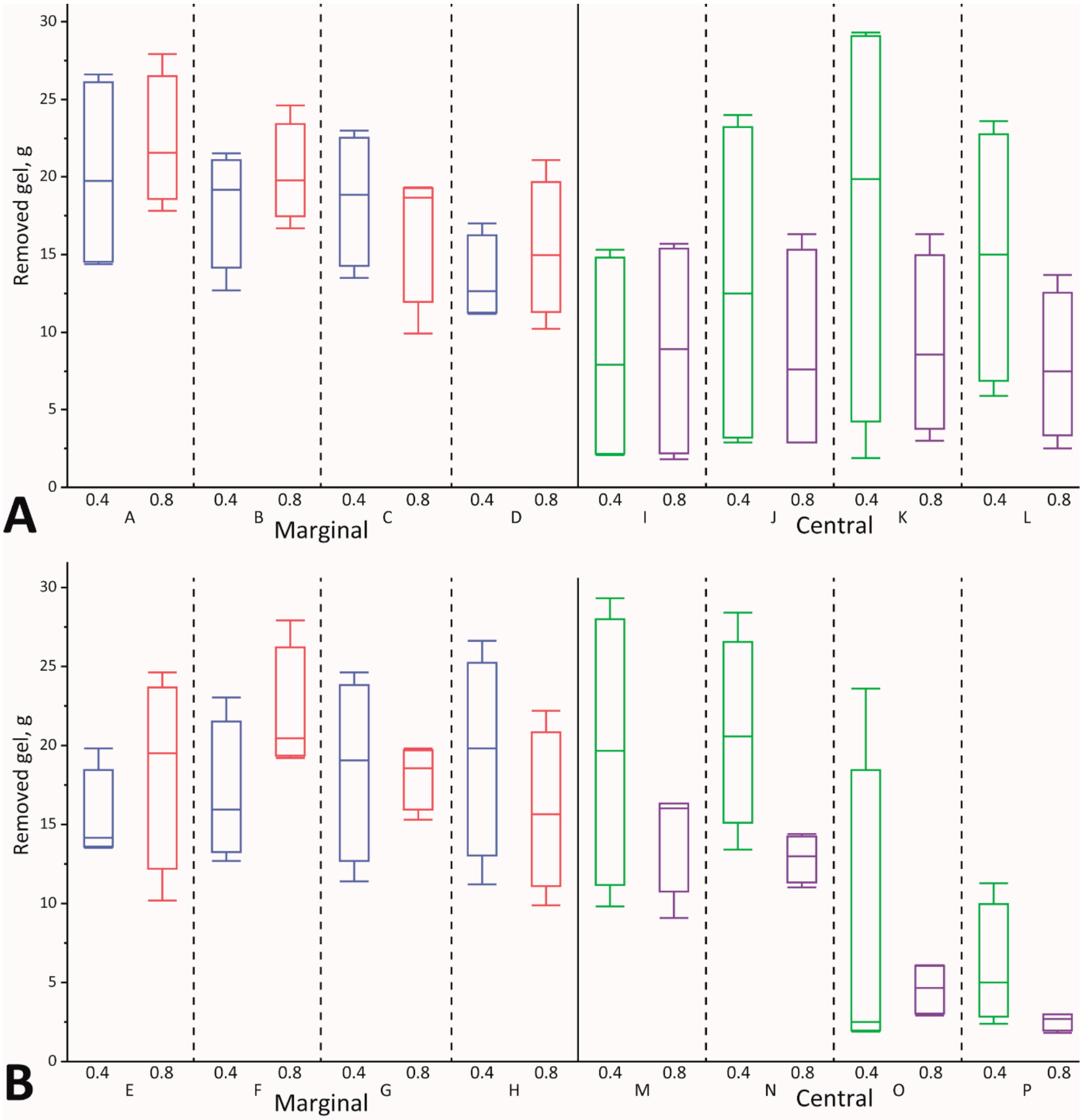

Supplementary Figure S20. Pull-through test results illustrating the effect of stylus (A) and cusp (B) morphology on feeding performance. For each tooth type, the removed mass of agar gel, given in g, is shown for the soft (0.4%) and stiff (0.8%) substrates, highlighting morphology- and substrate-dependent differences in material removal.

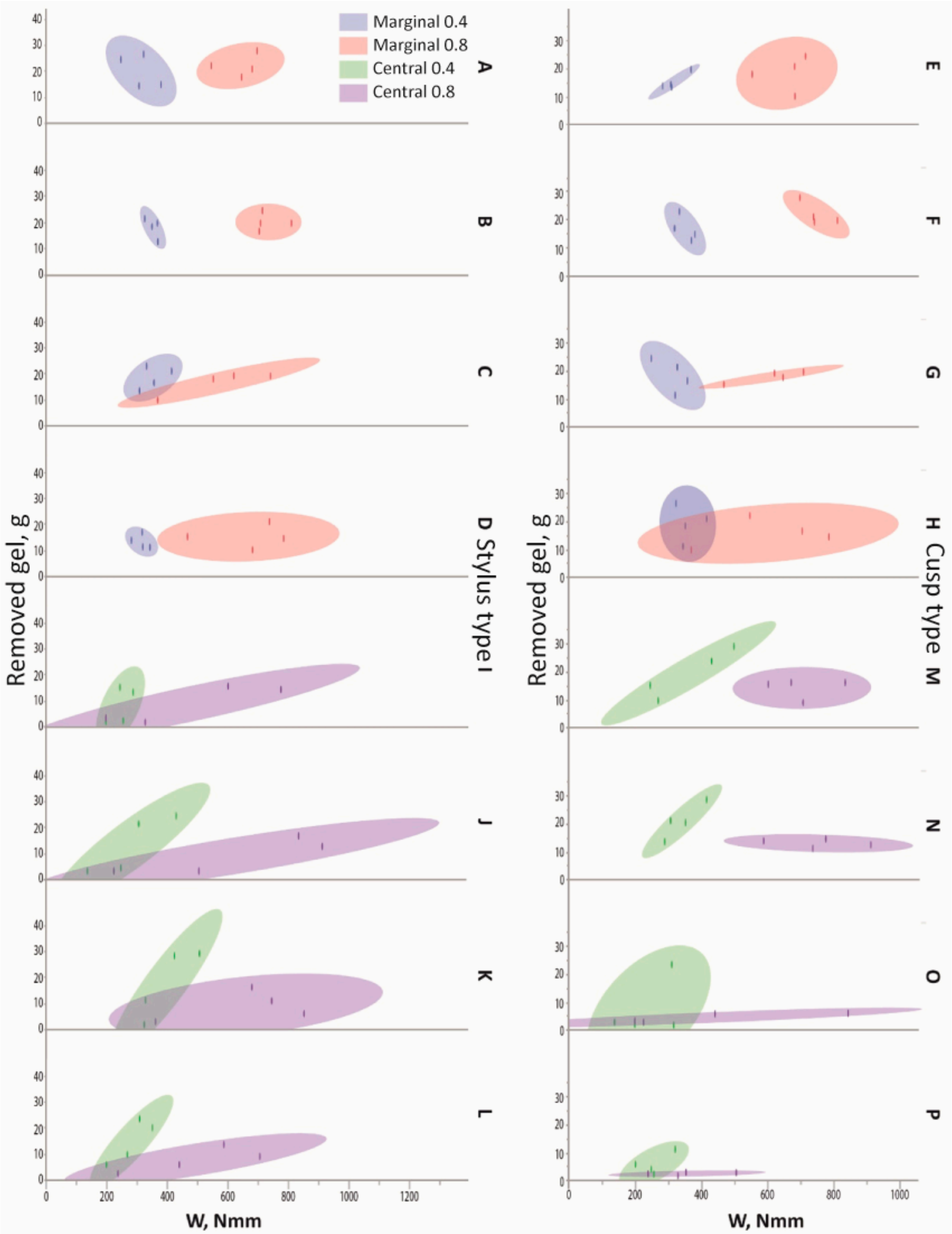

Supplementary Figure S21. Pull-through test results showing the relationship between removed gel mass, given in g, and mechanical work, given in Nmm, for the different tooth types in the two gels. Data are grouped by stylus types (left) and cusp types (right).

Supplementary Table S5. Ranking of central tooth efficiency separated by gel type, sorted from least to highest efficiency. Efficiency was calculated as mean work divided by removed mass. Lower values indicate higher efficiency, reflecting a lower energetic cost per unit of food collected.

0.4 % gel				0.8 % gel			
Model number	Removed gel, g	Mean work, Nmm	Efficiency Nmm/g	Model number	Removed gel, g	Mean work, Nmm	Efficiency Nmm/g
7	1.9	144.37	75.98	1	1.8	169.64	94.24
5	2.1	111.27	52.99	2	2.9	255.52	88.11
1	2.4	120.99	50.41	7	6.1	404.22	66.27
2	4.1	123.93	30.23	3	3.0	191.65	63.88
6	2.9	68.00	23.45	4	2.5	127.65	51.06
4	5.9	97.38	16.51	6	2.9	125.27	43.20
3	11.3	159.99	14.16	8	5.9	237.57	40.27
16	9.8	124.31	12.68	16	9.1	362.82	39.87
9	13.4	158.23	11.81	10	12.3	451.06	36.67
14	24	216.74	9.03	11	11.0	388.30	35.30
12	20.2	178.94	8.86	5	3.4	108.50	31.91
15	29.3	248.65	8.49	14	13.6	421.17	30.97
13	15.3	125.91	8.23	9	14.4	409.18	28.42
10	20.9	159.00	7.61	12	13.7	289.47	21.13
11	28.4	215.01	7.57	15	16.3	329.65	20.22
8	23.6	149.39	6.33	13	15.7	301.95	19.23

Supplementary Table S6. Ranking of marginal tooth efficiency separated by gel type, sorted from least to highest efficiency. Efficiency was calculated as mean work divided by removed mass. Lower values indicate higher efficiency, reflecting a lower energetic cost per unit of food collected.

0.4 % gel				0.8 % gel			
Model number	Removed gel, g	Mean work, Nmm	Efficiency Nmm/g	Model number	Removed gel, g	Mean work, Nmm	Efficiency Nmm/g
4	11.2	174.15	15.55	16	10.2	337.06	33.05
2	12.7	178.85	14.08	4	14.6	384.47	26.33
8	11.4	158.71	13.92	10	16.7	348.71	20.88
5	14.9	195.13	13.10	2	19.8	385.90	19.49
11	13.5	161.04	11.93	15	19.2	369.88	19.26
3	16.6	181.90	10.96	7	9.9	183.40	18.53
1	14.4	150.91	10.48	9	17.8	323.15	18.15
7	21.1	214.64	10.17	5	19.8	353.01	17.83
16	13.9	140.24	10.09	12	21.1	374.69	17.76
10	18.5	183.58	9.92	14	19.8	348.64	17.61
12	17.0	167.55	9.86	1	20.9	334.65	16.01
14	21.5	166.81	7.76	8	15.3	239.10	15.63
15	23.0	169.38	7.36	3	19.3	301.61	15.63
13	26.6	171.85	6.46	6	24.6	369.55	15.02
6	27.9	179.57	6.44	11	18.1	260.74	14.41
9	24.6	124.66	5.07	13	22.2	283.63	12.78

Supplementary Table S7. Summed efficiency values of the two gel types, providing an overview of the overall performance of each tooth variant across both substrate conditions. Sorted from least to highest efficiency. Variants with the lowest summed efficiency values performed best on both gel types and therefore rank highest. Efficiency was defined as work per removed mass, such that lower values indicate higher efficiency and higher values indicate less efficient use of work. Summing efficiency values across gel types allows direct comparison among tooth variants and experimental groups. Values were calculated from the individual efficiencies reported in Supplementary Tables S5 and S6.

Central tooth		Marginal tooth	
**Model number**	**Efficiency Nmm/g**	**Model number**	**Efficiency Nmm/g**
1	144.66	16	43.13
7	142.25	4	41.88
2	118.34	2	33.57
5	84.90	5	30.92
3	78.04	10	30.80
4	67.57	8	29.55
6	66.64	7	28.70
16	52.56	12	27.61
8	46.60	15	26.63
10	44.28	3	26.59
11	42.87	1	26.49
9	40.22	11	26.33
14	40.00	14	25.37
12	29.99	9	23.22
15	28.71	6	21.46
13	27.46	13	19.24
